# The COVID-19 pandemic in francophone West Africa: from the first cases to responses in seven countries

**DOI:** 10.1186/s12889-021-11529-7

**Published:** 2021-08-02

**Authors:** E. Bonnet, O. Bodson, F. Le Marcis, A. Faye, N. E. Sambieni, F. Fournet, F. Boyer, A. Coulibaly, K. Kadio, F. B. Diongue, V. Ridde

**Affiliations:** 1 Résiliences / PRODIG, French National Research Institute for Sustainable Development, 32 Avenue Henri Varagnat, 93140 Bondy, France; 2 Faculty of Social Sciences, University of Liège, Place des Orateurs 3, 4000 Liège, Belgium; 3 Triangle (UMR 5206), ENS de Lyon, TransVIHMI (UMI 233), French National Research Institute for Sustainable Development, Lyon, France; 4 Institut de Santé et Développement (ISED), Université Cheikh Anta Diop, Dakar, Senegal; 5 Faculty of Letters, Arts and Human Sciences (FLASH) and Laboratoire de recherches sur les dynamiques sociales et le développement local (Lasdel), University of Parakou, Parakou, Benin; 6 MIVEGEC (Univ Montpellier, IRD, CNRS), French National Research Institute for Sustainable Development, 911 Avenue Agropolis, BP 64501, 34394 Montpellier Cedex 5, France; 7 Research Unit “Migration and Society”, French National Research Institute for Sustainable Development, Associated with the Study and Research Group on Migration, Spaces and Societies, Abdou Moumouni University, Niamey, Niger; 8 Faculty of Medicine and Odontostomatology, University of Sciences, Techniques and Technologies, Bamako, Mali; 9 Institute for Health Science Research (IRSS), Ouagadougou, Burkina Faso; 10 Institute of Research for Development, Ouagadougou, Burkina Faso; 11 Centre Population et Développement (Ceped), IRD, French National Research Institute for Sustainable Development and Université de Paris, Inserm ERL 1244, 45 rue des Saints-Pères, 75006 Paris, France; 12 Institut de Santé et Développement, Université Cheikh Anta Diop, Dakar, Senegal

**Keywords:** COVID-19, SARS-CoV-2, West Africa, Intervention, Public health, Spatial analyses

## Abstract

**Background:**

In early March 2020, the COVID-19 pandemic hit West Africa. In response, countries in the region quickly set up crisis management committees and implemented drastic measures to stem the spread of the SARS-CoV-2 virus. The objective of this article is to analyse the epidemiological evolution of COVID-19 in seven Francophone West African countries (Benin, Burkina Faso, Côte d’Ivoire, Guinea, Mali, Niger, Senegal) as well as the public health measures decided upon during the first 7 months of the pandemic.

**Methods:**

Our method is based on quantitative and qualitative data from the pooling of information from a COVID-19 data platform and collected by a network of interdisciplinary collaborators present in the seven countries. Descriptive and spatial analyses of quantitative epidemiological data, as well as content analyses of qualitative data on public measures and management committees were performed.

**Results:**

Attack rates (October 2020) for COVID-19 have ranged from 20 per 100,000 inhabitants (Benin) to more than 94 per 100,000 inhabitants (Senegal). All these countries reacted quickly to the crisis, in some cases before the first reported infection, and implemented public measures in a relatively homogeneous manner. None of the countries implemented country-wide lockdowns, but some implemented partial or local containment measures. At the end of June 2020, countries began to lift certain restrictive measures, sometimes under pressure from the general population or from certain economic sectors.

**Conclusion:**

Much research on COVID-19 remains to be conducted in West Africa to better understand the dynamics of the pandemic, and to further examine the state responses to ensure their appropriateness and adaptation to the national contexts.

**Supplementary Information:**

The online version contains supplementary material available at 10.1186/s12889-021-11529-7.

## Background

There has been a split between ‘Afro-pessimists’ and ‘Afro-optimists’, with regards to the potential spread of the Sars-CoV-2 coronavirus. However, since the diagnosis of the first African case in Egypt on 16 February 2020 and the subsequent announcement of the pandemic by the World Health Organization (WHO) on 11 March 2020, little work has so far been published in scientific journals on the situation in Africa [1, 2]. As early as February 2020, initial modelling correctly estimated that the importation of the SARS-CoV-2 virus into Africa would first affect Egypt, as well as Algeria and South Africa [3]. At that time, researchers predicted that Francophone West African countries were at low risk of virus importation due to limited air traffic with China [3]. Africa was expecting to be well-prepared, according to members of the *Africa Centres for Disease Control and Prevention (*CDC Africa) [4], by the time the epidemic arrived. Four months after these estimates were made, it remains true that, compared to the rest of the world, the West African region does not seem to have suffered a major epidemic shock, especially if we compare the current situation with that experienced by some countries during the Ebola crisis [5, 6]. The most recent models predict that the 22% of the population in the African continent could become infected with SARS-CoV-2 during the first year of the pandemic, with approximately 150,000 deaths [7] and with peaks of contamination varying from one country to another [8]. The same researchers predict that Francophone West African countries will have few deaths related to the virus during the year: fewer than 800 in Benin, 1000 in Burkina Faso and just over 2200 in Senegal [7]. It has been estimated that the peak of cases in Senegal would occur between 28 May and 15 June 2020 [8]. However, these are all estimates and, at the time of writing this article, we still have little data on the reality of the state of the epidemic in Africa [9, 10] in contrast with the countries that were first affected [11, 12] nor do we have ample data on the effect of public health measures, which have been adapted in various ways to their national contexts [1, 4, 9, 13, 14]. As of 21 July 2020, CDC Africa estimates that there were 736,288 cases of COVID-19 in Africa and 15,418 deaths, representing only 5% of all reported cases worldwide. In Africa (54 countries), the case-fatality rate (CFR) has been reported at 2.1%, compared to a 4.2% average CFR in all countries where data are available (*n* = 215). West Africa alone accounts for 14.8% of cases and 11.2% of deaths on the African continent [15].

Faced with the academic divide between the Afro-optimists who believe that too much has been said about the fragility of health systems in Africa, and the Afro-pessimists who remind us of the disasters caused by the Ebola virus [16, 17], we would like to propose a third way, that of Afro-realism. In order to do so, we describe and analyse the situation in seven Francophone West African countries where our team members are established. The governments of all these countries did not wait until they were overwhelmed by the pandemic, nor did they wait for the call for public health measures and physical distancing from the WHO on 7 April 2020, to react [18]. Anticipating the arrival of SARS-CoV-2, each government quickly put public measures in place to counter the advance of the virus, even if their populations did not always fully support them. A survey carried out in early April 2020 in 20 major African cities showed that 30% of people were opposed to closing markets, 29% to stopping traffic between cities and 22% to closing places of worship [19]. In Senegal, on the other hand, a survey conducted in early April 2020 showed that 72.5% of people were in favour of a two-week lockdown and 85.6% were very or rather confident in the government’s capacity to deal with the crisis [20].

In addition, voices have been raised to question the lack of inclusion and equity in the governance bodies governing the management of the crisis and the public measures taken [21]. Similarly, amid rumours of the exploitation of the virus for political purposes and the ineffectiveness of public health measures and/or treatment, these issues were also being hotly debated in the public sphere. In Niger, for example, some believed that the pandemic was used for financial reasons: “*politicians are manipulating data to present more positive cases in the hope of winning funding from donors*” [22]. In Cameroon, people also question the statistics “*The death numbers from COVID19 is wrong*” [22]. Although the situation differed between countries, and our previous analyses have showed that routine data could be valuable for evaluating public health interventions in West Africa [23, 24], while the quality of health data in this region of the world is often debated and brought into question [25]. Frequently side-lined by the debates regarding the epidemiological data, public action against COVID-19 remains understudied [2]. In early June, a first study in Kenya with a sample of 213 people demonstrated the effectiveness of the policy package on the epidemic’s reproductive rate [26]; however, there has been a lack of similar analysis in the Francophone West African region. The objective of this article is to describe and analyse the epidemiological evolution of COVID-19 in seven Francophone West African countries during the first 7 months of the pandemic, as well as the public measures taken to deal with it.

## Methods

This paper analyses quantitative and qualitative empirical data from several sources, mainly collected from a regional platform on COVID-19.

### COVID-19 data platform

We launched *Covid19Africa.com*, designed as an information platform to track the SARS-CoV-2 pandemic, on 31 March 2020. Focusing on West Africa (including non-Francophone countries) and Francophone countries throughout Africa, the site is designed to facilitate contextual readings and effective management of data. The objective of the site is to facilitate the open and free sharing of data with a comparative perspective. The platform relies on a network of 26 contributors spread across the countries. A total of 32 countries are subject to daily monitoring of epidemiological data published on WHO recognized sites, official government sites and situation reports (SITREPs). All these public data are compared in order to validate the recognized situation in the countries. International sites were not preferred for this analysis because they publish data that often differ from the actual situation in these countries. Indeed, these websites/databases often consider the official case announcement date as the date of diagnosis, while there may be several (up to 3) days of difference between a positive case diagnosis and the official announcement of it. This is particularly the case for data relating to Benin and Burkina Faso.

### Study area

The analysis presented in this article focuses on seven West African countries (Benin, Burkina Faso, Côte d’Ivoire, Guinea, Mali, Niger and Senegal), which were chosen because they illustrate the varying dynamics of the pandemic and the taking of various government measures in the same geographical area, and because our team is sufficiently familiar with these contexts to analyse the respective national situations.

### Quantitative analyses

The epidemiological data from this study comprise the cumulative number of daily cases of COVID-19, the daily number of deaths and the number of tests performed. The study period is from 28 February 2020, the date of the first case detected in west africa (Nigeria), to 1 October 2020. The epidemic curves were constructed to describe and compare the trends in each country and a 7-day moving average was applied.

The data are dynamically mapped to visualize the distribution of cases and deaths, using a *Json* program developed by our team that allows the publication, in cartographic form, of country data entered into a shared database. Spatial analyses in the form of bivariate maps [27] and spatiotemporal analyses complement the dynamic maps available at *Covid19Afrique.com*. The bivariate maps combine the attack rate and the number of COVID-19 cases. The spatial and temporal analyses were performed using the spatial scan statistics implemented in SatScan (version 9.4) [28]. This method detects regions with higher-than-expected disease incidence in time and space by assigning them a relative risk, producing as a result a list of spatiotemporal clusters that can be used to identify the epidemic phases in the study area.

We propose an analysis of spatiotemporal clusters only for the countries where lower administrative data are available: Burkina Faso and Senegal, in order to illustrate the concentration of COVID-19 cases within countries, as well as the intermittent emergences of clusters within countries. We used Kulldorff’s space-time scan statistical analysis to detect the temporal, spatial, and space-time community clusters of COVID-19 at infra-national scale to verify whether the geographic clustering [28] of COVID-19 has been caused by random variation or not.

The system uses a spatio-temporal scan statistic in the form of a circle/cylindrical scan [29]. Space-time scan statistics complement baseline disease rate maps, and, using a variety of data models, can be used to determine whether observed space-time patterns of a disease are due to chance or randomly distributed. Scan statistics detect clusters that are outliers (a cluster not observed under baseline conditions). The statistics use cylinder (scan windows) that are centred on grid points and move (scan) systematically across a study area to identify clusters of cases (each window counts the number of cases aggregated by geographical unit) [30]. A retrospective analysis was carried out to identify all significant clustering events (epidemic phase) that occurred up until the time of writing this paper. The permutation model of the scanning statistics tests only used case data within each candidate cylinder and calculated the ratio between the number of observed cases (we considered that at least 5 cases of COVID-19 were needed to be considered) and the number of expected cases under the null hypothesis that the observed cases are randomly distributed in space and time. The expected number of cases was calculated as the sum of all observed cases multiplied by the size of the scanning window and divided by the size of the entire study area [28]. The observed/expected ratio (Tables 3 and 4) was used to estimate the probability that a candidate cylinder represents a true significant clustering event of COVID-19 cases. The window with the maximum likelihood is defined as the most likely cluster area, and other clusters with statistically significant log-likelihood ratios (LLR) were defined as the secondary potential clusters. The *P*-values of LLR were estimated through 999 Monte Carlo simulations. We have mapped the attack rates of COVID-19 per commune in order to display on the same map both the global situation of the epidemic and the clusters detected by this analysis. The cartographic documents thus summarize the epidemic situation in the outliers.

Some spatial and temporal analyses have already been carried out in Africa on the influence of meteorological factors in promoting or hindering the spread of the aerosol pathogen COVID-19 in Africa [31] or on the early spatial and temporal dynamics of COVID-19 in the first 62 days of the disease outbreak on the African continent [32]. This analysis adds to those already published by Adekunle [31] and Gavawam [32] but this paper is one of the first spatio-temporal analysis carried out in African countries at lower administrative level, but is part of a trend including other similar studies carried out in the USA at the beginning of the pandemic [30] and a study in Kuwait targeting a specific population within the capital [33].

These analyses are, however, dependent on the implementation of the tests in the regions and on the reporting modalities, but they do make it possible to highlight situations where the local incidence is high for all localities, especially smaller ones. The reporting process is identical in Burkina Faso and Senegal, which makes the analyses comparable.

Spatial cluster analyses were performed using SaTScan® v9.4.4 (Martin Kulldorff, Harvard Medical School, Boston, MA, USA and Information Management Services Inc., Silver Spring, MD, USA. The maps were generated using Quantum-GIS® v3.10 (Open Source Geospatial Foundation Project, Beaverton, OR, USA).

### Qualitative analyses

A documentary analysis based on situation reports from country ministries, scientific articles, reports from the WHO, CDC Africa, and the national press was compiled to enable the recording and tracking of events and government measures to produce the synthesis of information presented in this article. A qualitative analysis of the content of these documents was carried out in addition to a situational analysis carried out by the researchers present in each of the seven countries. A transversal analysis of the content of these studies was carried out and validated by all the authors of this article.

## Results

### The epidemiological situation at the end of September 2020

Although the seven countries do not collect data in exactly the same way, and while it is unclear exactly what are the differences are, we found it interesting to compare trends in the number of cases and the number of deaths in an overall way that has evolved over the study period.

**Fig. 1:**
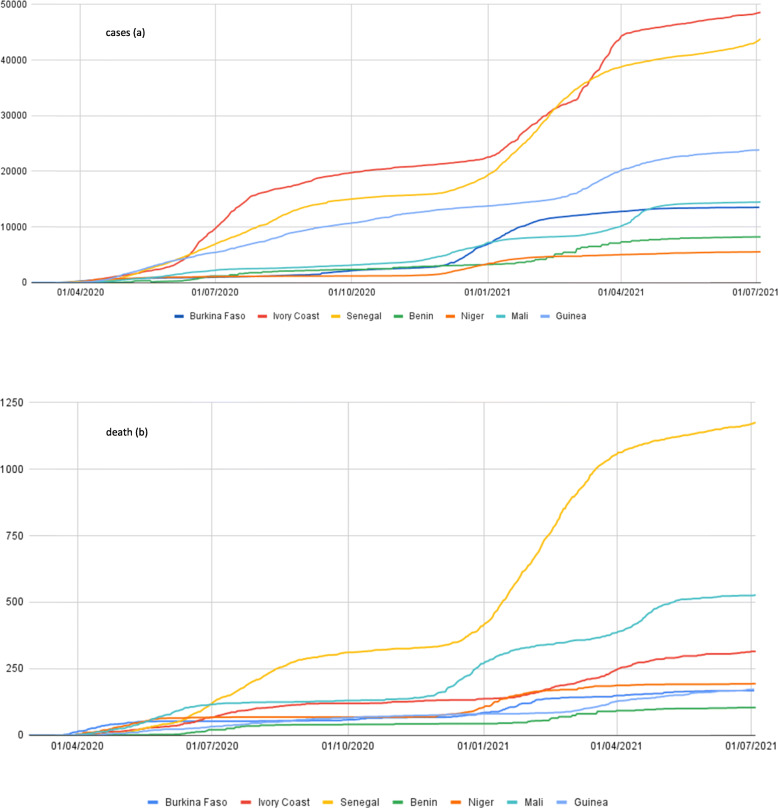
Cumulative COVID-19 cases (**a**) and deaths (**b**) by country between 28 February and 1 October 2020

The first cases in the ECOWAS zone were identified on 28 February 2020 in Nigeria, followed by Senegal, Burkina Faso, Niger, Mali and Ghana during the month of March. Measured according to identified case rates, there are three groups of countries: Nigeria and Ghana with more than 10,000 registered cases, followed by Senegal, Guinea and Côte d’Ivoire with 5000 cases and the rest of the ECOWAS countries with fewer than 1000 cases (Fig. 1). Deaths caused by the virus, which have been few in Africa, remain below 100 in all countries except Nigeria.

**Fig. 2:**
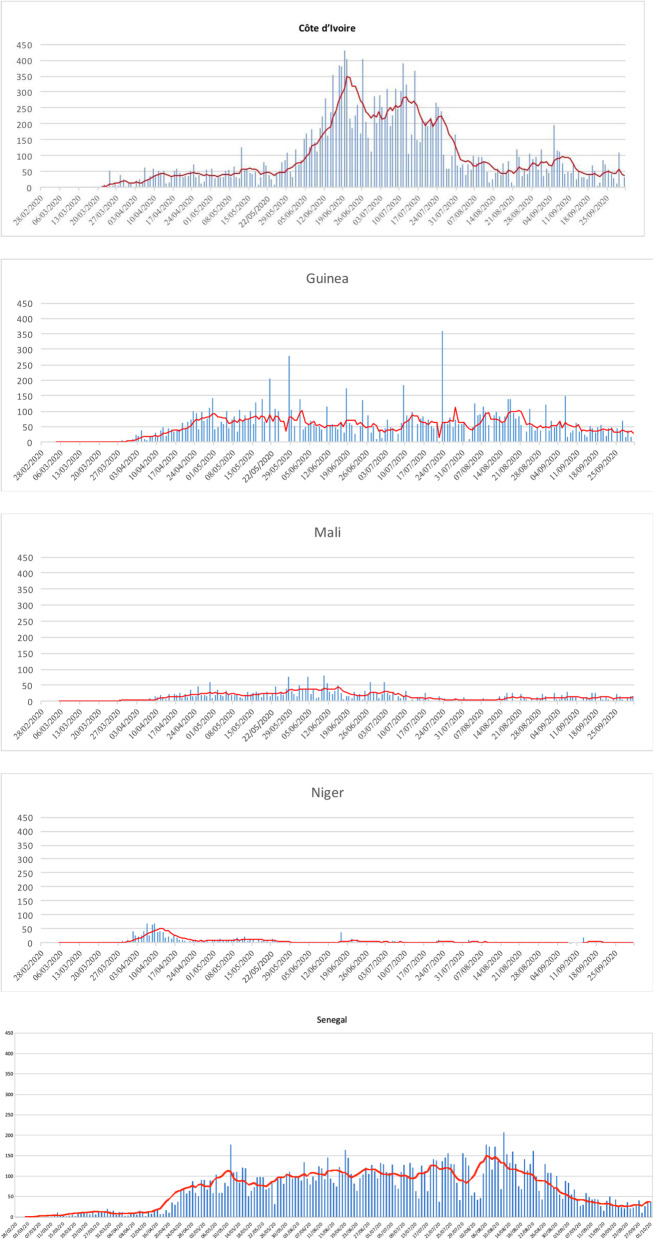
Number of daily cases and moving average (7 days) per country (red line) between 28 February 2020 and 1 October 2020

During the month of April, all seven countries were registering many cases and entering an ascending phase (see Additional file 1 for a figure with a country-specific scale). At the end of April, Guinea, Côte d’Ivoire and Senegal entered an intense ascending epidemic phase, while Burkina Faso and Niger reached their peak (Fig. 2). Since the beginning of May, the epidemics in Burkina Faso and Niger have begun to decline, based on the number of reported cases. By mid-June, both countries had several consecutive days with zero cases detected. Over the same period, Guinea and Senegal appeared to have reached a plateau, but both countries still had a significant daily number of cases. Benin and Côte d’Ivoire showed a very different pattern, with a doubling of cases every 3 days. While for Benin, it is important to put this situation into perspective as the number of cases remains low (Fig. 2 uses the same scale for the seven countries), for Côte d’Ivoire, the situation is more complex.

**Fig. 3:**
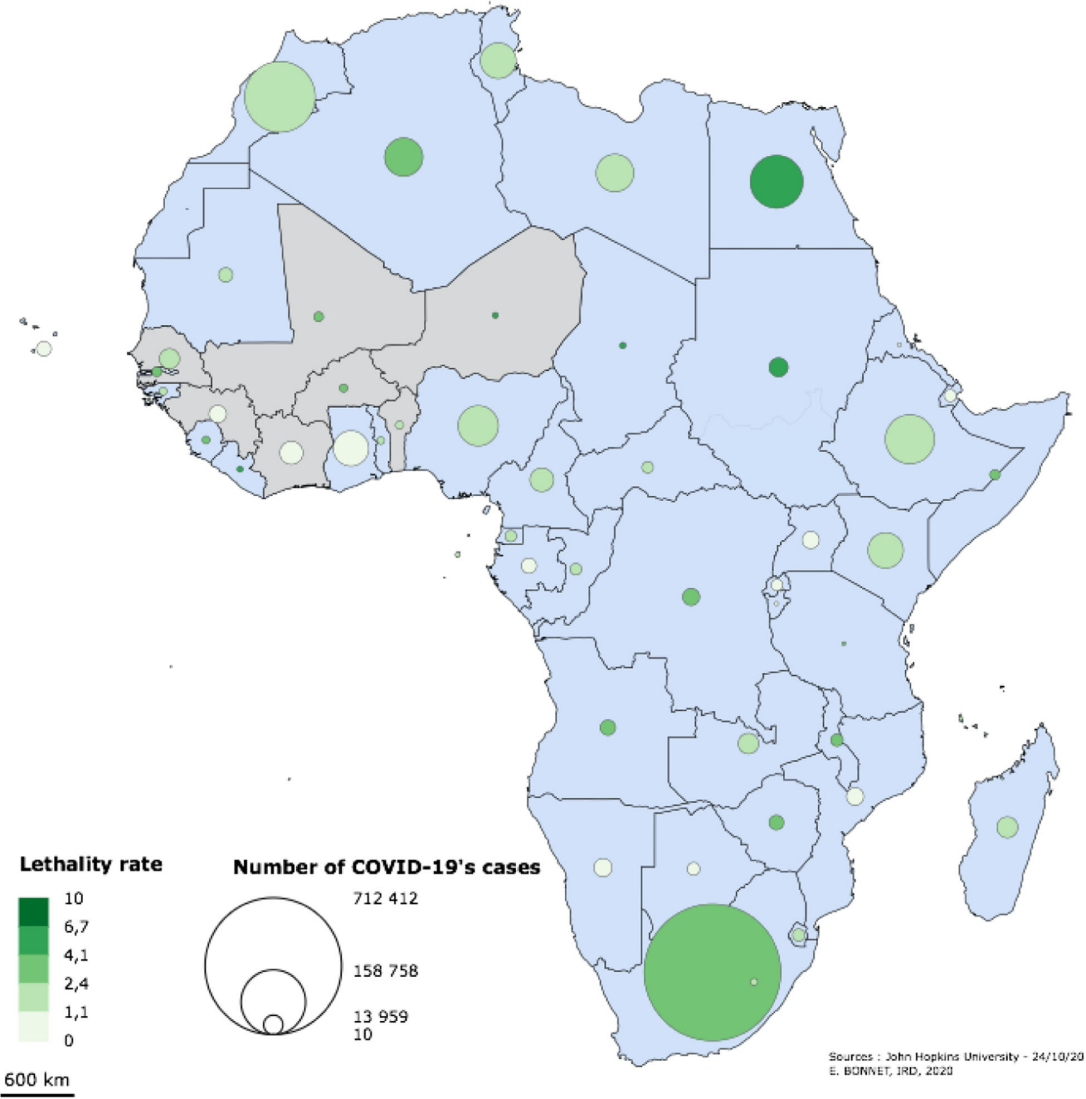
Case and Case-Fatality Mapping in Africa (in brown, country of our study)

During the same period, COVID-19 attack rates ranged from less than 5 per 100,000 inhabitants (Niger) to more than 95 per 100,000 inhabitants (Senegal). The three Sahelian countries (Burkina Faso, Mali, Niger) experienced the highest case-fatality rates. In Benin, Côte d’Ivoire and Senegal, the case-fatality rate remains low; however, the epidemic had not yet started to decline by the end of October 2020. Throughout the period, Guinea and Benin experienced comparatively lower rates (Table 1 and Fig. 3).

**Table 1:** Attack rate and Case Fatality rate of COVID-19 (end of February to 1 October 2020)

	Benin	Burkina Faso	Côte d’Ivoire	Guinea	Mali	Niger	Senegal
Attack rate per 100,000 population	20,51	10,20	78,79	85,82	15,65	5,33	94,75
Case fatality rate	1,73	2,73	0,60	0,61	4,18	5,76	2,07

All of these results depend, however, on the detection of cases, and therefore on the number of tests performed, which is highly variable (Table 2). In Burkina Faso, Guinea, Mali and Niger, the low number of tests, which can only be performed in some regional capitals, probably explains a higher case-fatality rate than elsewhere. However, it should be noted that *testing* data are not always available at sub-national level and have only been available since the end of March in most of the target countries. Benin is a special case, as it is the only country to have been forced to revise its COVID-19 statistics. On 19 May, the country was in fact asked to revise the figures for positive cases from 339 to 130 because 209 people had been declared positive with a rapid diagnostic test (RDT), which the WHO did not recognize as valid. Despite the data revision, the country continued to apply the existing protocols and to treat active cases among the 209 people diagnosed by RDTs. Moreover, it was only by the end of June that RDT data were no longer published by the Beninese government, though RDTs continue to be used for travellers arriving at Cotonou airport.

**Table 2:** Number of PCR tests per 100,000 inhabitants as of 1 October, 2020

Benin	Burkina Faso	Côte d’Ivoire	Guinea	Mali	Niger	Senegal
1458	247	606	52	263	113	1177

#### Spatio-temporal analysis

**Fig. 4:**
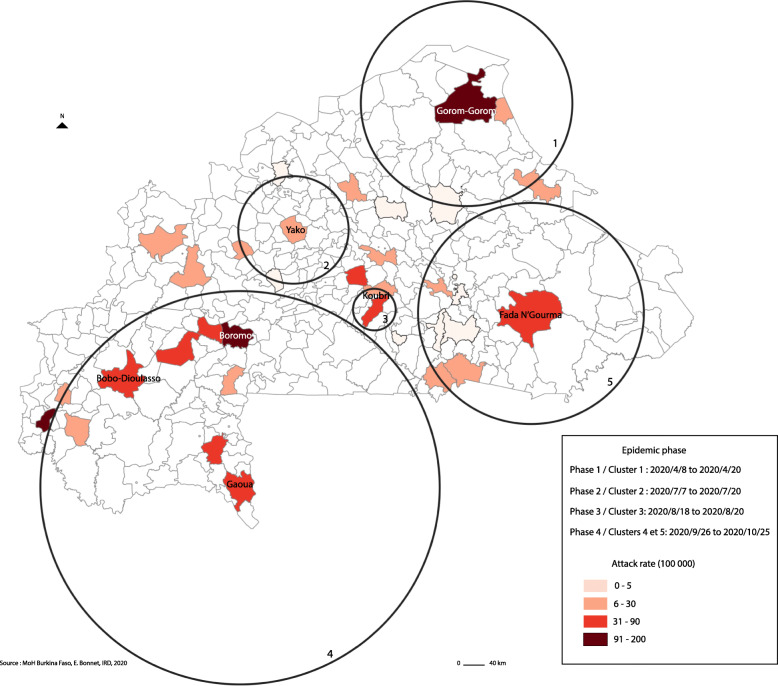
Spatio-temporal cluster of COVID-19 – Burkina Faso

Spatio-temporal analysis displayed five significant high-risk spatio-temporal clusters in Burkina Faso (Fig. 4, Table 3). These five clusters illustrate four temporal phases (phases 3 and 4 include two clusters at the same period), between April and October 2020, where higher than expected concentrations of cases occurred. The size of the circles, measured with the radius of the circle in kilometres, represents the most likely cluster area. The first occurred during the month of April in the Gorom-Gorom region (cluster 1), after the first cases were detected in Ouagadougou in early March 2020. The second phase occured in July after measurements were relaxed, in Yako (Cluster 2) with a very high observed/expected ratio over a short period. The third phase occurred in August and was concentrated a few kilometres south of Ouagadougou in Koubri Cluster 2). Finally, the last phase (Clusters 4 and cluster 5) referred to two regions, in the Southwest and East of Burkina Faso, and took place between September and October, after the air borders had been reopened and the traffic associated with the return of summer breaks over the period in the country and the capital city.

**Table 3:** Emerging space-time cluster of Covid-19 in Burkina Faso

Cluster	Duration	*p*	Observed/expected	Radius
1	2020/4/8 to 2020/4/20	< 0.001	10,13	133 km
2	2020/7/7 to 2020/7/20	< 0.001	43,64	70 km
3	2020/8/18 to 2020/8/20	< 0.001	33,94	28 km
4	2020/9/26 to 2020/10/25	< 0.001	1,97	256 km
5	2020/9/26 to 2020/10/25	< 0.001	6,99	144 km

**Fig. 5:**
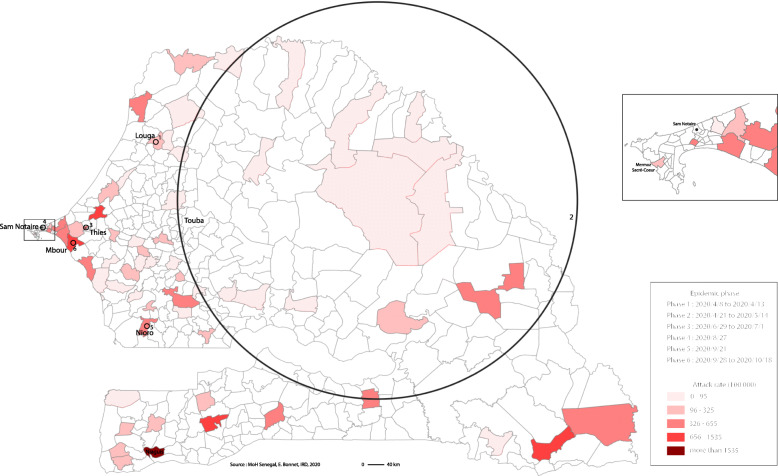
Spatio-temporal cluster of COVID-19 – Senegal

Spatio-temporal analysis displayed six significant high-risk spatio-temporal clusters in Senegal (Fig. 5, Table 4). These six clusters illustrate 6 temporal phases, between April and October 2020, where higher than expected concentrations of cases occurred. The first occurred during the month of April in the Louga region, after the beginning of the epidemic in early March 2020. The second phase appeared at the end of April, and continued until mid May in a large region around Touba (maximum likelihood). The third phase occurred at the end of June in Thies. The fourth phase took place in August around Dakar (Sam-Notaire), while the fifth phase began at the beginning of September at Nioro and the last phase (sixth) occurred in Mbour in October.

**Table 4:** Emerging space-time cluster of Covi-19 in Senegal

Cluster	Duration	*p*	observed/expected	Radius
1	2020/4/8 to 2020/4/13	< 0.001	147,71	10 km
2	2020/4/21 to 2020/5/14	< 0.001	3,51	219 km
3	2020/6/29 to 2020/7/1	< 0.001	6,26	10 km
4	2020/8/27	< 0.001	6,15	10 km
5	2020/9/21	< 0.001	44,13	10 km
6	2020/9/28 to 2020/10/18	< 0.001	8,94	10 km

As there is a long time between the writing and the publication of an article, we propose an update of the data in the Additional file 4 (tables and maps as of July 3, 2021). All countries have since been subjected to a second wave, or even a third for some countries (Côte d’Ivoire, Mali and Senegal), see https://www.covid19afrique.com.

### State responses to the COVID-19 pandemic

#### Government measures against the pandemic

All the countries analysed have planned, and subsequently implemented, several government measures.

Strong government measures were first rapidly implemented. They were then gradually strengthened and targeted, before being reduced or even lifted.

##### Rapid and strong primary government measures

Many countries prepared the pandemic response and organized actions either before or overlapping with the time of diagnosis of the first national cases (Table 5). In the course of March 2020, we witnessed a rapid implementation of measures to control travellers. Guinea and Senegal formulated even more restrictive responses: both countries have introduced a curfew, facilitated by the declaration of a state of emergency. Nevertheless, none of the countries analysed has yet implemented large scale lockdowns throughout their national territory as has been done elsewhere in the world. However, borders between each region were closed very quickly.

**Table 5:** Date of First Case and First Significant Government Measure in 2020

Country	Date of first case	Date of measure	Nature of the first significant government measure
**Benin**	16th March	1st March	Border temperature control
**Burkina Faso**	9th March	3rd March	Prohibition of national and international events
**Côte d’Ivoire**	13th March	4th March	Establishment of a response plan focusing on epidemiological and biological surveillance, prevention, management of potential patients, information and public awareness of compliance with COVID-19 prevention measures
**Guinea**	12th March	25th January	Systematic check-ups upon arrival of travellers at International airport (temperature, hand sanitizer, health questionnaire) and at the port of Conakry.
**Mali**	25th March	19th March	Prohibition of gatherings Closure of schools and universities
**Niger**	21st March	13th March	Prohibition of gatherings Self-isolation on return from abroad
**Senegal**	2nd March	14th March	Prohibition of gatherings

##### Gradual intensification and targeting of government measures

Following the rapid first government measures, the trend towards increased strictness continued into April and, partially, into May 2020. During this period, there was a significant reinforcement of bans everywhere, which became increasingly drastic, despite the fact that the number of cases has remained very low and that the trends had not been exponential (Fig. 2). Among these strict measures, it is worth noting the obligation to wear masks, the enforced reduction in the number of people on public transport and the closure of markets etc.

This response quickly intensified as the virus gained ground. On 10 April 2020, Mali launched the “one Malian, one mask” programme and the President of the Republic announced that he had ordered 20 million washable masks.

Capitals and large cities have not only been the scene of primary care for the sick. In some countries (Burkina Faso, Côte d’Ivoire, Niger), they were also the target of restrictive measures, as the data on the evolution of the pandemic seemed to show that it particularly affected areas of these countries where large populations are concentrated. For example, in Niger, the wearing of masks for employees and users of public and parastatal services were only made compulsory in Zinder on 5 May, while in Niamey, the capital, which has also been subjected to sanitary isolation, mask-wearing has been compulsory in urban transport, markets, supermarkets, shops and public squares since April 9. In Côte d’Ivoire, the 10 communes of the capital were isolated from the rest of the country on March 25. Many countries have banned travel between regions (Guinea, Côte d’Ivoire, Senegal) without specific authorization, with an exception for the transport of goods. This is notably the case in Benin where a *cordon sanitaire* isolating the South from the rest of the country was established on March 30.

##### Gradual reduction or lifting of government measures

The months of May and June 2020 have been part of a trend towards the easing or even lifting of initial nationwide measures, such as the reopening of cultural venues in Senegal on 11 May 2020 and the reopening of schools in Niger on 1st June 2020. For some countries, the process of lifting these measures has been somewhat hasty, not to say improvised. In Senegal, the announcement of the reopening of schools for examination classes scheduled for 2 June 2020 was cancelled the day before, instead being postponed until 25 June 2020. The Senegalese President, who was himself under quarantine, announced the lifting of the state of emergency and curfew on 29 June for the following day, with a reinstatement of office hours from 8 a.m. to 5 p.m. However, places hosting leisure activities behind closed doors remained closed, as did public markets. In Côte d’Ivoire, such prevarication has also been present, for example, regarding the end of the *cordon sanitaire* around Greater Abidjan scheduled for 31 May 2020, and finally extended until 14 June 2020 and then lifted on 15 July to allow, according to some, the funeral of the Prime Minister to take place.

#### Six challenges of government measures against the pandemic

Taking government measures in response to the COVID-19 pandemic raised several challenges in the contexts under review.

The first challenge identified concerns measures surrounding places of worship. The decisions in many Francophone West African countries (Benin, Burkina Faso, Côte d’Ivoire, Guinea, Senegal) to close places of worship has, in some cases, led to tensions between governments and religious representatives (albeit not in Côte d’Ivoire). For example, in Senegal, the closure was highly contested and the decision was quickly lifted. Although some Catholic representatives decided to keep their churches closed, the Muslim community reacted in a heterogeneous way, with many places of worship remaining open. In Mali, on the other hand, where links between the government and clerics have been highly controversial since 2009, due largely to tense debates on the ‘family code’ within the legal system, and where the government has been discredited by poor management of the recent security crisis, the overwhelming majority of mosques remained open, and many Muslim leaders did not hesitate to speak out against the suspension of prayers in these places of worship. The argument, relayed notably by the radio stations, is that one should not be afraid of illness and therefore ‘attack God’ by not attending prayers. They have argued, on the contrary, that collective prayers help to eradicate the disease. In Guinea, the establishment of two-tier measures has been astonishing: mosques have been closed while markets remain open.

The second challenge relates to centralisation decisions and in particular the centralisation of organisation of care. Most countries have been setting up sites or structures specifically dedicated to patient care, although many began by initially centralising all medical care in capitals and large, densely populated cities. In Côte d’Ivoire, both the analysis of diagnostic tests and the treatment of patients were initially carried out only in Abidjan; the university hospital in Bouaké - another important city in the country - was not operational for tests until the end of May, two and a half months after the first case in the country. In Burkina Faso, only the capital and the second largest city in the country initially had treatment centres. In Guinea, the epidemic treatment centres in the interior of the country were not yet operational as of mid-April and the laboratories first able to detect cases were located in Conakry and in Kindia. Senegal has innovated by very quickly organizing contact tracing and management for COVID-19 positive cases, quarantining suspected cases in hotels. However, the country was quickly overwhelmed by the lack of space, and the government was criticized by some hoteliers for not paying for their services. A process of decentralization of care was then organized for asymptomatic or mild cases in dedicated non-hospital sites (Senegal, Guinea). It was then decided at the end of June 2020 to no longer systematically test the contacts of positive cases and to limit the tests to symptomatic and vulnerable persons.

The third challenge relates to the social acceptability of restrictive public health measures. The measures taken by governments have sometimes given rise to protest movements, the scale of which varied depending on the context, questioning the veracity of the pandemic, refuting certain state measures considered disproportionate in view of the other health problems that these countries encounter or judged inappropriate for a pious population (Niger), denouncing their catastrophic socio-economic repercussions (Burkina Faso) or the way public forces racketed the population under the pretext of implementing prevention measures (Guinea).

The fourth challenge relates to the decisions making processes based on the existence of evidence and social contestations. Although the role played by popular protests on the lifting of certain restrictive measures has not been refuted, other reasons, frequently political and socio-economic, also explain the easing of state measures. The health-based justifications for lifting these measures seem to have taken little account of the epidemiological curves, which did not change dramatically during this period. For example, in Benin, Côte d’Ivoire or Senegal, Fig. 2 clearly shows that the trends did not change, with or without measurement. For these three countries and for the others, it is as if there was no logical connection between the evolution of reported cases and government measures. This lack of logic is sometimes compounded by a lack of consistency in the measures taken.

The fifth challenge concerns the logic and coherence of certain measures. In Guinea, until the beginning of April, the measures were disorganised and lacked coherence. This was largely the product of thinly-veiled competition between the director of the ‘Agence Nationale de Sécurité Sanitaire’ (National Health Security Agency) and the Minister of Health, partly as a result of the importance of this agency in the fight against Ebola, largely supported by donors, working in silos and involving little coordination with the Ministry of Health. We have seen, for example, some replications of the response to the Ebola crisis with the implementation of the ‘Stop Covid in 60 Days’ plan, a replica of the ‘Stop Ebola in 60 Days’ plan, which marked the end of the epidemic with its “micro-circling” strategy. In addition, the significant development of diagnostic capacities in Guinea, a benefit induced by the Ebola epidemic, has created a negative consequence in the fight against the pandemic: the abandonment of the community approach and community care. In Côte d’Ivoire and Senegal, for example, we have seen that the lifting of travel restrictions for teachers returning to their home regions, for example, has had the immediate consequence of the virus being transmitted by these individuals, especially in transmission-free areas. In Mali, the government decided to maintain the second round of legislative elections on 19 April while the epidemic was spreading throughout Africa.

The sixth challenge relates to reconciling political, business and public health priorities. In all of the target countries, the coinciding of the electoral schedule and the epidemic implies a necessarily political reading of the measures put in place. Moreover, it would seem that it has also been the social and economic consequences of policies that have pushed most countries to reduce or scale down their health measures. Indeed, economic measures were taken very quickly to support households or businesses, for example by postponing the payment of water and electricity bills (Côte d’Ivoire, Burkina Faso, Guinea), by reducing the price of fuel (Guinea), by exempting electricity and water bills from value added tax (Mali), by subsidising the tourist industry (Senegal) or by organising a vast distribution of food to the poorest households (Côte d’Ivoire, Mali, Senegal). Senegal has, for example, created a ‘Fund for the Fight against the Effects of COVID-19’ (“FORCE-COVID-19”) to be granted an endowment of CFA 1000 billion (1.5 billion Euros).

#### National Health Response Plans

We analysed and compared the health response plans of Burkina Faso, Côte d’Ivoire, Mali, Niger and finally Senegal, excluding Benin due to a lack of data. The national response plans were mostly devised following the first diagnosed cases of COVID-19 in their respective national territories. Most of them were launched between March and April 2020. However, Senegal stands out, however, because it had a response plan in place before the first case of COVID-19 was detected on its soil. It is also the only country to have indicated the period of application of its plan, which officially ended in July 2020.

Countries defined the overall objective of their response plan as enabling them to have the capacity to respond to or control the pandemic. Only Senegal and Mali raise (timidly) the ethical issues associated with the response. The countries have developed their response plan around activities that refer to seven major dimensions: 1) planning, coordination and monitoring, 2) epidemiological surveillance, case investigation and entry point controls, 3) laboratory (biological surveillance), 4) prevention and infection control measures, 5) risk communication (health education) and community engagement/mobilization, 6) case management (including health system strengthening) and 7) evaluation and research (Table 6). However, all these dimensions have not been developed at the same stage of response to the pandemic and do not represent the same financial burden between countries (Additional file 2).

**Table 6:** Percentage of budget associated with the different health activities in individual country plans

	Burkina Faso	Côte d’Ivoire	Guinea	Mali	Niger	Senegal
1.Planning, coordination and monitoring	78,4%	*	*	2,4%	4,3%	3,3%
2.Epidemiological surveillance (including case investigation and port of entry controls)	5,5%	*	*	33,5%	36,7%	12,3%
3.Biological monitoring (laboratory)	0,2%	*	*			4,2%
4.Infection prevention and control measures	9,4%	*		6,4%	4,6%	19,7%
5.Risk communication and community engagement	0,6%	*	*	6,3%	30,7%	13,4%
6.Case management (including health system strengthening)	5,7%	*	*	51,4%	22,2%	47,1%
7.Evaluation and research	0,2%	*			1,5%	
TOTAL	100%	*			100%	100%

Note: *: data not available

The budgets for response plans vary also widely between countries (Table 7). The response plan budget is around $16.20 per capita in Burkina Faso compared to $0.10 per capita in Niger.

**Table 7:** Health budget of response plans by population size, by country ($ USD)

	Burkina Faso	Côte d’Ivoire	Guinea	Mali	Niger	Senegal
« Health » budget of response plans	320,246,961.5	172,584,037.1	114,617,250	6,071,250.6	2,618,839.3	2,593,034.4
Budget for health component only, per habitant	16,2	6,9	9,2	0,3	0,1	0,2

However, the availability of budgets is not guaranteed, even though the majority of countries do not mention this fact in their documents. In Burkina Faso, the country announced in the plan that it had released a financial package of 500 million CFA francs ($890,592), or 0.28% of its budget. Furthermore, the country announced that 2.41% of its plan was covered by external contributions that had already been pledged, of which slightly less than 10% (9.6%), i.e. 412,958,116 CFA ($735,554.4) had already been released, which raises the question of the effective implementation of these plans.

#### Crisis management committees

The analysis of the various committees formed in the context of the pandemic in the seven countries is a challenge, given the lack of transparency in the national communications on their creation and implementation. In addition, there are several sub-committees and commissions that revolve around the primary bodies, the outlines of which are not always very clear. There seem to be two main groupings: on the one hand, the bodies managing the response to the pandemic and, on the other hand, the consultative bodies. The bodies behind the creation of these different committees are either ministries or, as in the case of the Monitoring Committee for the implementation of the operations of the FORCE COVID-19 created in Senegal, the presidency or head of government. In Senegal, the setting up of a scientific committee was announced, but it appears never to have been organized; nevertheless, we have seen the establishment of research and ethics commissions. The bodies identified by our analysis (Additional file 3), were mostly created following the first cases of SARS-CoV-2. The composition of the bodies identified depends primarily on their mandate; the committees whose mandate is oriented towards surveillance and research objectives are mostly composed of scientists, while the members of bodies with a mandate for response management are most often from the public sector, including a significant number of ministers. Finally, we have observed the numerical importance of scientists from the basic sciences, a significant under-representation of women, the rare presence of technical and financial partners and the notable absence of actors from the voluntary sector, civil society, patient representatives, and from the private sector. Guinea is an exception here, however, with a scientific committee chaired by a woman, a gynaecologist, and two vice-chairmen, an anthropologist and a virologist. It should also be noted that there are many commissions within the Agence Nationale de Sécurité Sanitaire (National Health Security Agency) that work on specific themes and welcome NGO actors (communication commission, laboratory commission, etc.) and a inter-ministerial committee for the fight against the coronavirus epidemic 19 headed by the prime minister.

## Discussion

Supported by our team of multidisciplinary collaborators from the COVID-19 data platform, we have undertaken this project to describe and analyse the epidemiological evolution in seven Francophone West African countries during the first 7 months of the pandemic, and the public measures taken to deal with it. Our epidemiological analysis demonstrated the diverse nature of COVID-19 outbreaks depending on the context of its spread, and has highlighted the delicate issue of case detection.

However, despite the diversity of contexts and epidemiological situations in the countries [2], we have noticed a certain similarity in the reactions to the arrival of the pandemic among them. It is true that each country was able to take advantage of the relatively late arrival of the virus in the sub-region, compared to the Asian and European continents, in order to prepare and even anticipate certain health measures. It has been hypothesized that this was also due to the use of evidence, including advice from WHO and the Africa Centre for Disease Control [34]. Conversely, other measures seem to have been taken in haste and without consultation, which have led to misunderstandings, frustration and protests. Studies have been undertaken on the social acceptability of the measures [35] to be carried out, but the mistrust often encountered and the sometimes violent demonstrations (Côte d’Ivoire, Guinea, Mali, Niger, Senegal) show that the decisions behind the measures and their content have not always been understood and integrated into policy. A certain form of inconsistency, as elsewhere in the world, has also been noted regarding the lifting of certain restrictive measures, the reasons for which are probably other than health concerns. This lack of consistency between epidemiological curves and public health measures has thus sometimes led to scepticism about the very existence of COVID-19 (not as world pandemic, but as a reality in the specific case of African countries discussed here), as was sometimes the case with Ebola [36]. However, anticipation and preparation are precisely at the heart of epidemic management as the case of Ebola has clearly shown in the region [37] and trust and governance are essential elements of good pandemic preparedness [37].

Also, and despite the respite offered by the gradual advance of the pandemic, we have found that countries took a number of relatively similar - if not identical - health measures, which more fundamentally raises the question of the appropriateness of these measures to their national contexts and reawakens the myth of a “turnkey” response applicable to all and at all times. Yet all the scientific literature on public health interventions, including in Africa [38, 39] and on COVID-19 [13] affirms the importance of taking contexts into account in order for measures to be effective [40]. This need to contextualize the health response also requires taking into account the specificities of the disease. Although the state of knowledge on this virus is still limited and constantly evolving, evidence of the effectiveness or the processes to be used to develop or organise specific actions is already available. For example, regarding containment measures, a scoping review synthesising relevant knowledge published in 2018 highlights the importance of community involvement for their effectiveness [41]. A study on Ebola in 2014–2016 in the region similarly showed the need for community involvement in disease control interventions that take into account local dynamics [39]. The analysis of the situation in five African countries at the beginning of the pandemic also showed the importance of community involvement [10]. However, communities are still far from the process of reflection and formulation of health measures to be introduced within the context of COVID-19. This unfortunate observation has also been made for COVID-19 in Europe and elsewhere [21, 42]. However, community engagement will be undoubtedly essential when testing the eventual vaccine, as was the case for Ebola [43].

The observation drawn here is also valid for the management committees which, in the seven countries analysed in this paper, as elsewhere in the world [21], have effectively neglected to involve representatives of users, patients or NGOs. As everywhere, the power of these committees remains inexorably in the hands of clinicians, as the interdisciplinary, intersectoral or health promotion approach has been totally ignored [21]. Similarly, the presence of women has been completely side-lined, here as elsewhere, yet, as Bali et al. noted in a recent paper “*women are not only a vulnerable population, they can serve as agents of change whose contributions can improve epidemic response and recovery*” [44]. However, this situation of exclusion is deeply rooted in the region and the pandemic has therefore not been able to change this state of path dependency. The paradigm shift in public health approaches that this pandemic has shown to be indispensable is still far off [45].

As in most countries across the world, politics has also been widely invoked in the management of the pandemic in the countries of the region. This has been prevalent in countries where elections were held during the crisis (Benin, Guinea, Mali) but also in countries where political movements have taken advantage of some of the challenges faced by governments and political parties in power, in order to attack them in the face of upcoming elections (Burkina Faso, Côte d’Ivoire, Senegal). In Côte d’Ivoire, funds distributed by Deloitte to help large companies to counterbalance the economic crisis precipitated by the pandemic were offered primarily to companies sympathetic to the ruling party. As a result of such actions, several civil society organizations in many countries have sometimes denounced the state of “*management in total uncertainty*”, such as the National Coalition for Health and Social Action in Senegal. The same is true for religious bodies where, in some countries such as Senegal or Mali, they have taken a prominent place in the debates and had a major influence on political decisions concerning certain measures, particularly, but not exclusively, concerning the closure of places of worship (especially for Muslims in the context of the Ramadan period). The pandemic has thus sometimes again highlighted the close links between the religious and political spheres in the region.

Although some believe, as in South Africa, that the measures taken have made it possible to delay the peak of the epidemic [14], we believe that, in the context of the seven countries concerned here, such an assessment is impossible to make given the current state of knowledge. Driven by the urgency to act, measures have been applied almost everywhere and without any real means of evaluating their effectiveness. Moreover, most of these interventions were stopped, or their scope was reduced, at the end of June 2020, leaving a very short time window for evaluation, not to mention the fact that few research organizations will be able to measure the degree of fidelity with which they were implemented, and the real application of these measures in these countries [46]. A study in the Democratic Republic of Congo showed that the official recommendations to wear masks were not respected [47]. The challenges for modelling the effectiveness of these interventions will be enormous. In addition, these interventions were in most cases so complex that it is doubtful whether their effectiveness can be studied systematically [48, 49]. Yet we were warned a few years ago in the region that “*considering a public health measure with such dramatic social effects as containment, the transnational scientific community should engage rapidly in building evidence about the efficacy of containment in the Ebola outbreak*” [50]. Several countries in the region have implementing seroprevalence surveys, the results of which may shed more light on the circulation of the virus, the effectiveness of interventions on health outcome or simply the herd immunity.

Finally, our cross-sectional analysis confirms all the challenges related to data access and the importance of promoting open access to data, especially when, as is often the case in the region, access to documents and epidemiological data is particularly complicated and difficult. The COVID-19 pandemic has only confirmed the importance of this situation [2, 51] where no country in the region has yet put its epidemiological data, apart from those communicated daily to the media, online. Something that the West African Health Organization (WAHO) should do. The organization of our collaborative platform has made it possible to create this dynamic of rapid information sharing, as international or sub-regional organizations have not been able to achieve this speed of response. However, our analysis also highlights the challenges of the quality of these data, particularly when, for example, deaths are not counted in communities in Guinea in a disaggregated manner, when RDTs (Rapid Diagnostic Tests) are used in Benin or when the number of tests is reduced in Senegal or Guinea. The magnitude of the pandemic is thus likely to be underestimated here [52], as elsewhere [49]. Access to epidemiological data, if available, will make it possible to assess excess mortality in the countries, at different territorial scales, and thus estimate whether the figures disseminated reflect the real situation [47]. Similarly, the proposed infra-national analysis illustrates the association of the spread of the epidemic from the capitals to the secondary cities. The spatio-temporal analysis characterizes situations where the incidence is much higher than expected. It thus shows that on an infra-national scale, all territories and communities are impacted and exposed to the virus, but are also sometimes much more isolated from health care facilities than large urban centres. It is also important to consider here that the analysis is at the local level of the municipality where the tests are carried out. This is a less usual scale of analysis but one that allows even the smallest number of households to be identified with a high incidence. This type of analysis proves to be very useful in the epidemic phase for targeting affected areas, but requires good quality data that can be retrieved in real time, to do this, it is necessary to mobilize prospective studies in order to identify spatio-temporal clusters. In several aspects, COVID-19 thus demonstrates the fundamental need for credible data as a governance tool to identify and support populations, particularly the most vulnerable [53].

## Conclusion

The objective of this article has been to describe and analyse the situation of the COVID-19 pandemic and the state responses organized in seven Francophone West African countries. The comparative analysis identified recurrences in the contexts, public interventions and the reaction of social actors. Simultaneously, our analysis also shows that it is difficult to understand the dynamics of the pandemic in these contexts; COVID-19 is slowly spreading in the region, but it is circulating and is likely to continue to do so for a long time to come. The state of knowledge about this new coronavirus is still in an embryonic stage and research in this region of the world is still scarce. It is therefore becoming urgent and indispensable to mobilize interdisciplinary research teams to better understand the dynamics of the pandemic regarding the interventions implemented in order to ensure their appropriateness and effective adaptation to the contexts.

## Supplementary Information

**Additional file 1.** Updated Covid-19 data July 2021.

**Additional file 2.** Timing of activities, by component and country.

**Additional file 3.** Some Pandemic Monitoring and Management Bodies.

**Additional file 4.** Updated Covid-19 data (July 2021).

## Data Availability

The dataset(s) supporting the conclusions of this article are available at https://www.covid19afrique.com.

## Abbreviations

NGO: Non-Governmental Organization
RDT: Rapid Diagnostic Tests
WHO: World Health Organization
