# Supplementary material for: The COVID-19 pandemic in francophone West Africa: from the first cases to responses in seven countries
Source: BMC Public Health. 2021 Aug 2;21:1490. doi: 10.1186/s12889-021-11529-7 (PMC8327893; doi:10.1186/s12889-021-11529-7)
Supplement: Supplementary file 2 — Additional file 2. Timing of activities, by component and country. [file 12889_2021_11529_MOESM2_ESM.docx]

**Additional files 2: Timing of activities, by component and country**

|  |  | BF | Cameroun | CI | Guinée | Mali | Mauritanie | Niger | Sénégal | Tchad |
| --- | --- | --- | --- | --- | --- | --- | --- | --- | --- | --- |
| **Health component** | | | | | | | | | | |
|  | 1. Planning, coordination and monitoring | █ █ | * | * | * | █ | * | █ █ █ | * | █ █ |
|  | 2. Epidemiological surveillance (including case investigation and port of entry controls) | █ █ | * | * | * | █ | * | █ █ █ | * | █ █ |
|  | 3. Biological monitoring (laboratory) | █ █ | * | * | * |  | * |  | * | █ █ |
|  | 4. Infection prevention and control measures | █ █ | * | * |  | █ | * | █ | * | █ |
|  | 5. Risk Communication and Community Engagement/Mobilization | █ █ | * | * | * | █ | * | █ █ █ | * | █ |
|  | 6. Case management (including health system strengthening) | █ █ | * | * | * | █ █ | * | █ █ | * | █ █ |
|  | 7. Evaluation and research | █ █ | * | * |  |  | ^1^ | █ | ^1^ | █ |
| **Social component** | | | | | | | | | | |
|  | Household support |  |  |  | * |  | * |  |  |  |
|  | Support to vulnerable households |  |  |  | * |  | * |  |  |  |
|  | Promotion of human rights |  |  |  |  |  | * |  |  |  |
| **Economics component** | | | | | | | | | | |
|  | Support for the national economy |  |  |  |  |  | * |  |  |  |
|  | Support to the private sector |  |  |  | * |  | * |  |  |  |

█ during the pre/pre-epidemic phase (Scenario 1 for Chad)

█ during the epidemic/ (response phase for BF) (scenario 2 for Chad)

█ After the epidemic

*Presence of elements but inability to determine the period of application of activities associated with sub-components

^1^ Some evaluations are, however, listed in the Mauritania and Senegal plan, but they refer more to the coordination and monitoring dimension, and are not developed as a specific strategic axis.
