# Supplementary material for: The COVID-19 pandemic in francophone West Africa: from the first cases to responses in seven countries
Source: BMC Public Health. 2021 Aug 2;21:1490. doi: 10.1186/s12889-021-11529-7 (PMC8327893; doi:10.1186/s12889-021-11529-7)
Supplement: Supplementary file 3 — Additional file 3. Some Pandemic Monitoring and Management Bodies. [file 12889_2021_11529_MOESM3_ESM.docx]

**Additional files 3 : Some Pandemic Monitoring and Management Bodies**

| **Pays** | **Name** | **Creation date** | **Creative Authority** | **Profil** | | **Mandate** | | | **Number of members (including women)** |
| --- | --- | --- | --- | --- | --- | --- | --- | --- | --- |
|  |  |  |  | **Manageent** | **Consult./ Scienc.** | **Surv./ monitoring** | **Riposte** | **Rech.** |  |
| **Bénin** | Committee of Experts | April 2020 | Ministry of Education and Scientific Research |  | 🗶 |  |  | 🗶 | 23 (2) |
|  | Committee of Experts on COVID-19 | 01-04-20 | Ministry of Health |  | 🗶 |  |  | 🗶 | 13 (2) |
|  | Ad hoc inter-ministerial committee for the management of the health emergency linked to the outbreak at Covid-19 | NA | Ministries of Health, Foreign Affairs and Cooperation, Interior and Public Security, and Infrastructure and Transport | 🗶 |  | 🗶 | 🗶 |  | 4 (0) |
| **Burkina Faso** | National Covid Outbreak Management Committee-19 | 17-03-20 | Primature | 🗶 |  |  | 🗶 |  | 28 (NA) |
| **Côte d’Ivoire** | Health Action Unit | NA | NA | 🗶 |  |  | 🗶 |  | NA |
|  | Steering Committee | NA | NA | 🗶 |  |  | 🗶 |  | NA |
|  | Health Monitoring Committee | NA | NA | 🗶 |  | 🗶 |  |  | NA |
| **Guinée** | Scientific Council on Pandemic Response to Coronavirus Disease | 14-04-20 | Presidential Decree  Government Secretariat-General |  | 🗶 | 🗶 | 🗶 | NA | 17 (3) |
|  | Interministerial Committee for the Control of the Coronavirus Epidemic | 03-04-20 | Primature | 🗶 |  |  | 🗶 |  | 22 (3) |
| **Mali** | Crisis Committee for the Management of Coronavirus and Crimean Outbreaks Congo | 25-02-20 | Ministry of Health and Social Affairs | 🗶 |  |  | 🗶 |  | 12 (2) |
|  | Scientific Committee for Pandemic Coronavirus Management (covid-19) | 10-04-20 |  |  | 🗶 | 🗶 |  |  | 12 (1) |
| **Niger** | Inter-ministerial Committee for the Fight against the Coronavirus Pandemic | 25-03-20 | Primature | 🗶 |  |  | 🗶 |  | NA |
|  | Panel of Experts |  |  |  | 🗶 |  |  |  | 10 (1) |
|  | Consultative Committee |  |  |  | 🗶 |  |  |  | 13 (1) |
| **Sénégal** | Committee to monitor the implementation of the operations of FORCE Covid-19 (Response and Solidarity Fund against the Effects of Covid-19) | 17-04-20 | Presidency | 🗶 |  |  | 🗶 |  | 30 (NA) |
