# Supplementary material for: The COVID-19 pandemic in francophone West Africa: from the first cases to responses in seven countries
Source: BMC Public Health. 2021 Aug 2;21:1490. doi: 10.1186/s12889-021-11529-7 (PMC8327893; doi:10.1186/s12889-021-11529-7)
Supplement: Supplementary file 4 — Additional file 4. Updated Covid-19 data (July 2021). [file 12889_2021_11529_MOESM4_ESM.docx]

Appendix : Updated Covid-19 data (July 2021)


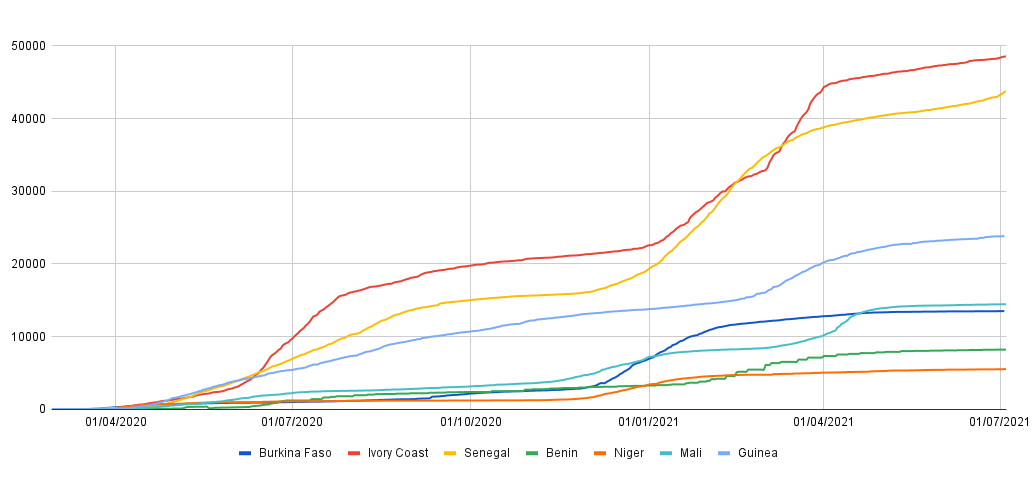


(a)


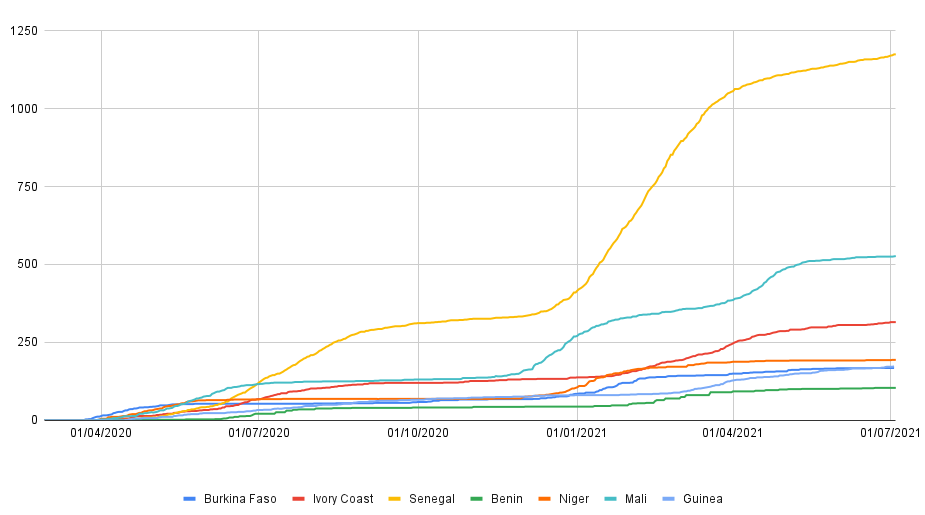


(b)

Figure 1: Cumulative COVID-19 cases (a) and deaths (b) by country between 28 February and 3 July 2021


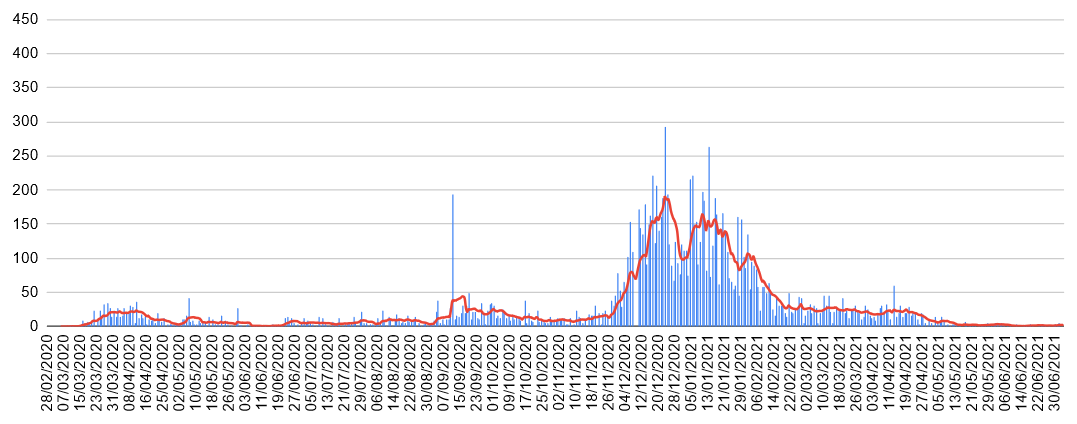


Burkina Faso


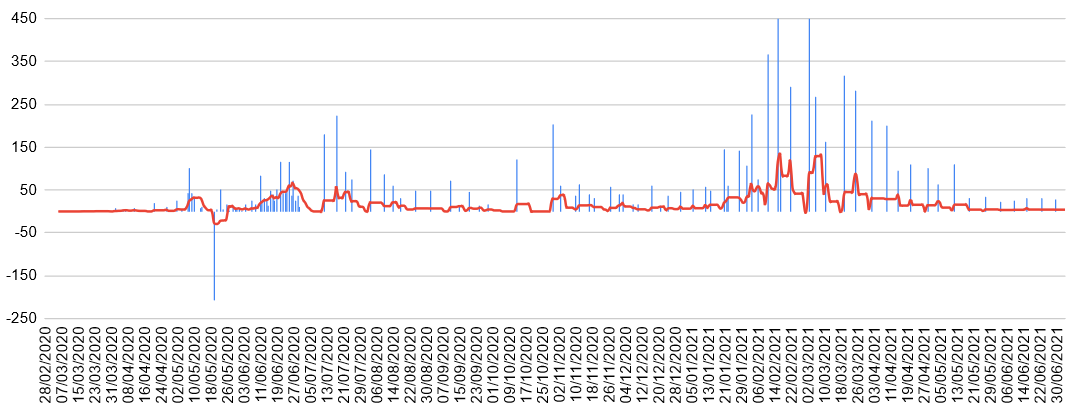


Benin


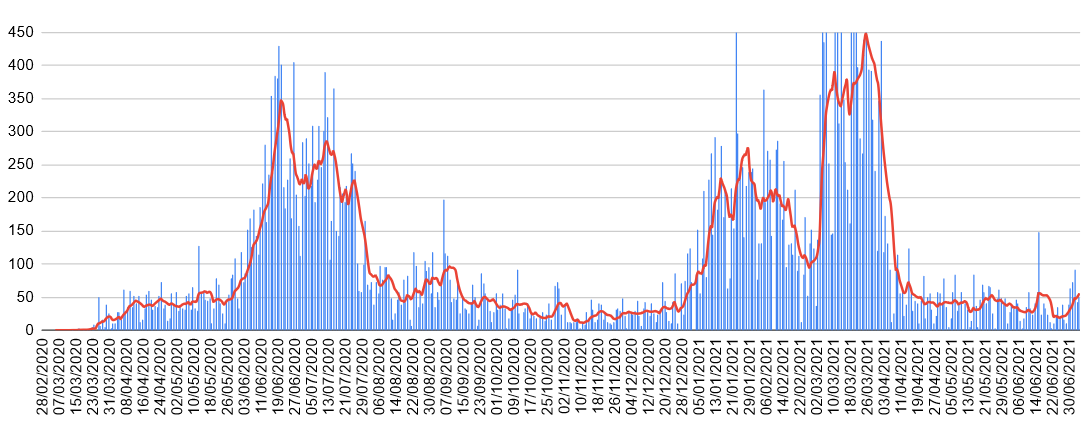


Ivory Coast


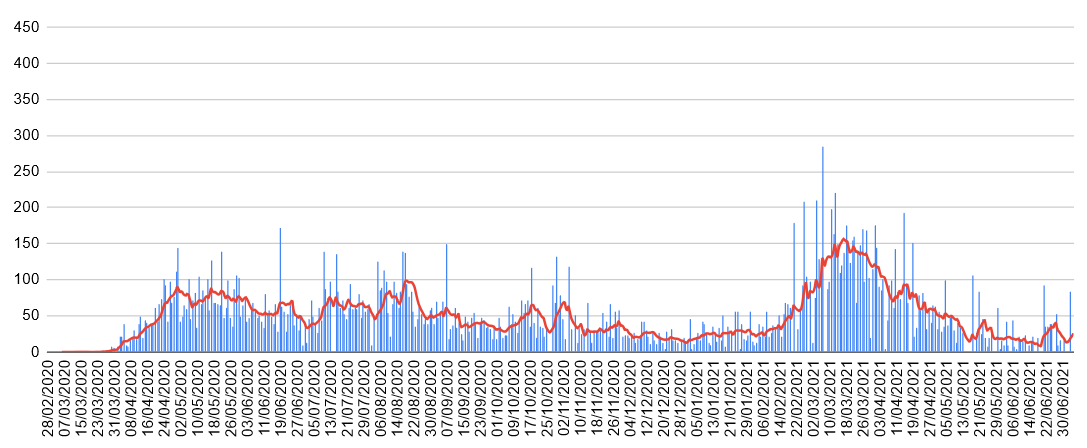


Guinea


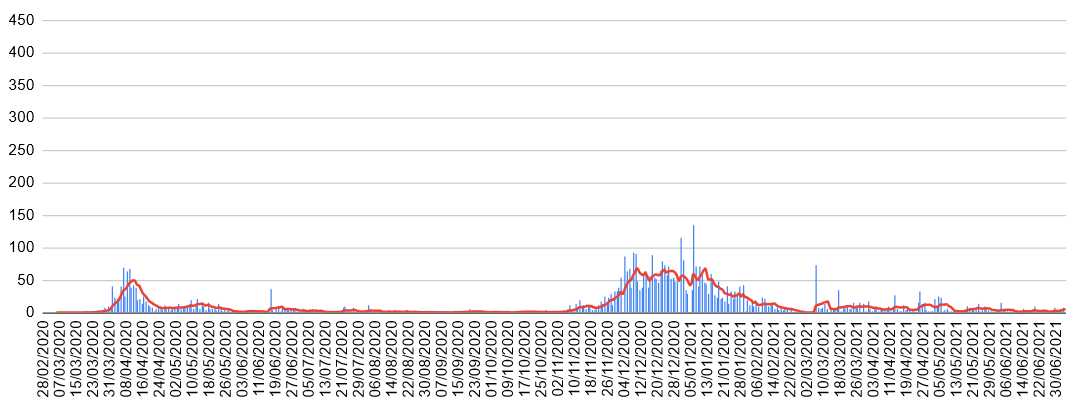


Niger


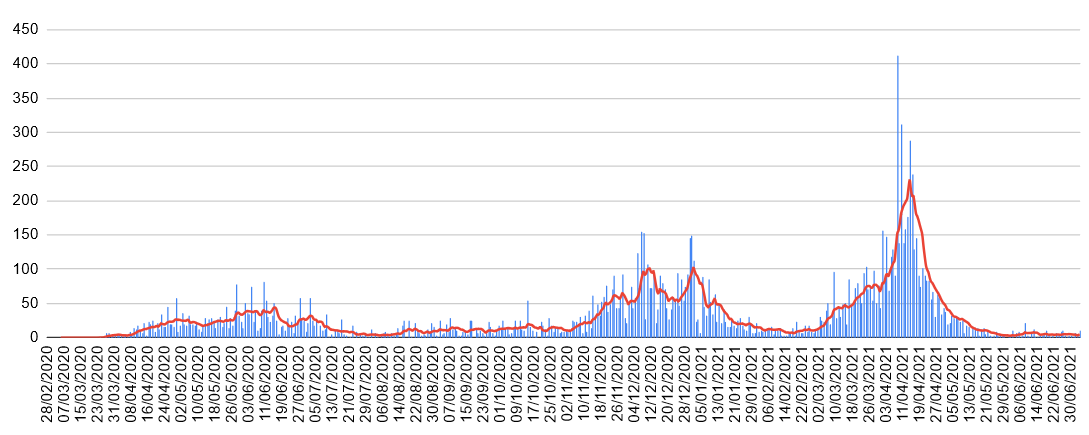


Mali


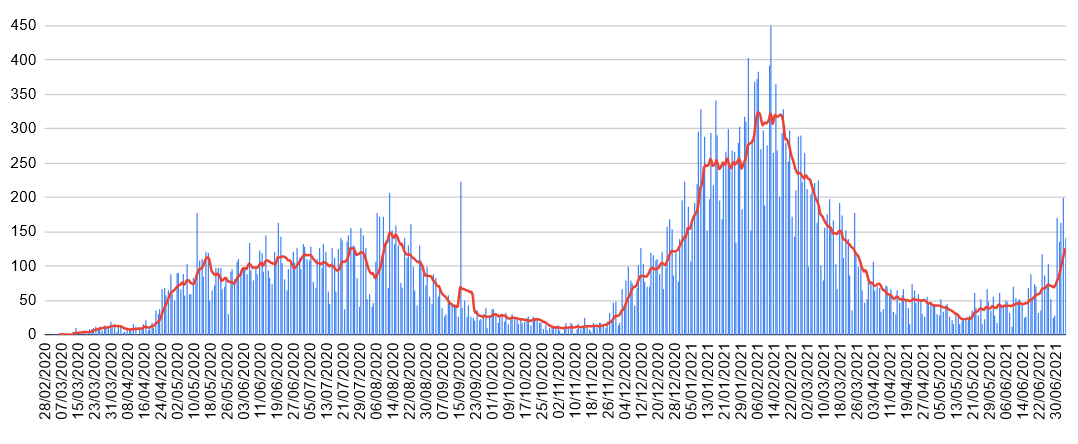


Senegal

Figure 2: Number of daily cases and moving average (7 days) per country (red line) between 28 February 2020 and 3 July 2021

Table 1: Attack rate and Case Fatality rate of COVID-19 (end of February to 3 July 2021)

|  | **Benin** | **Burkina Faso** | **Côte d’Ivoire** | **Guinea** | **Mali** | **Niger** | **Senegal** |
| --- | --- | --- | --- | --- | --- | --- | --- |
| Attack rate per  100,000 population | 71,32 | 62,73 | 183,12 | 179,80 | 68,97 | 22,80 | 269,15 |
| Case fatality rate | 1,27 | 1,24 | 0,65 | 0,77 | 3,64 | 3,53 | 2,69 |

Table 2: Number of PCR tests per 100,000 inhabitants as of 3 July, 2021

| **Benin** | **Burkina Faso** | **Côte d’Ivoire** | **Guinea** | **Mali** | **Niger** | **Senegal** |
| --- | --- | --- | --- | --- | --- | --- |
| Not available since June 2021 | 991 | 2703 | 2991 | 1529 | 428 | 360 |


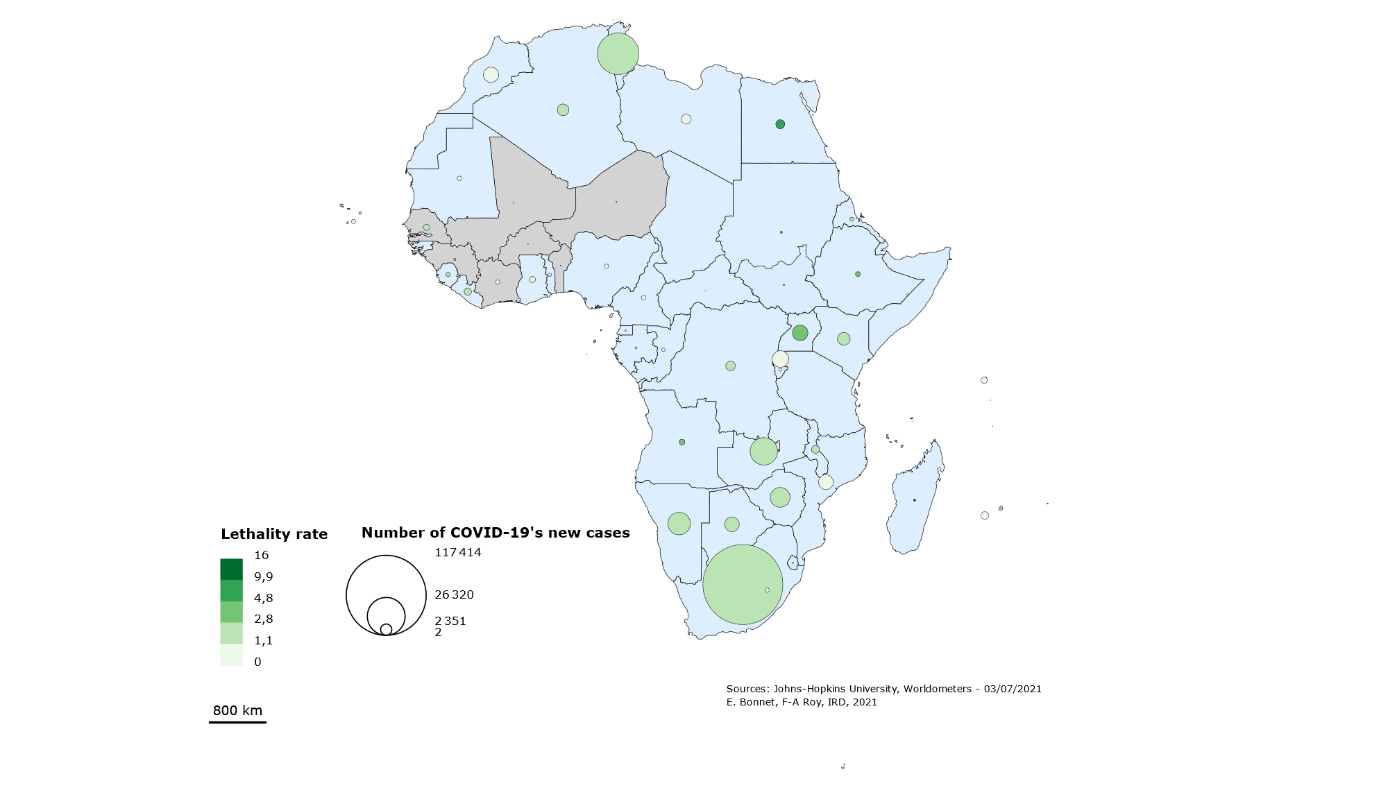


Figure 3: Case and Case-Fatality Mapping in Africa (in brown, country of our study)

(2020/03 – 2021/06)

**CLUSTERS DETECTED in Burkina Faso 2020/03/01 to 2021/06/30**

1.Location IDs included.: DIEBOUGOU, DANO, GAOUA, KARANGASSO VIGUE, HOUNDE, LENA, BOROMO,

BAGASSI, SIDERADOUGOU, BOBO DIOULASSO

Coordinates / radius..: (10.928009 N, 3.261135 W) / 114.18 km

Time frame............: 2020/9/11 to 2020/12/12

Number of cases.......: 70461

Expected cases........: 47722.74

Observed / expected...: 1.48

Test statistic........: 4815.279714

P-value...............: < 0.00000000000000001

2.Location IDs included.: GOROM GOROM, FALAGOUNTOU

Coordinates / radius..: (14.398626 N, 0.256172 W) / 46.57 km

Time frame............: 2020/4/10 to 2020/11/29

Number of cases.......: 5791

Expected cases........: 1663.79

Observed / expected...: 3.48

Test statistic........: 3098.538189

P-value...............: < 0.00000000000000001

3.Location IDs included.: BOGANDE, PIELA, MANNI, TOUGOURI, BOULSA, YALGO, GAYERI, SEBBA,

POUYTENGA, KOUPELA, ZORGHO, BOUROUM, DORI, KORSIMORO, KAYA, SEYTENGA,

BARSALOGHO, FADA, ZINIARE, TENKODOGO

Coordinates / radius..: (12.948564 N, 0.048381 W) / 137.14 km

Time frame............: 2020/12/21 to 2021/4/6

Number of cases.......: 73543

Expected cases........: 61206.52

Observed / expected...: 1.20

Test statistic........: 1196.281583

P-value...............: < 0.00000000000000001

4.Location IDs included.: KOMBISSIRI

Coordinates / radius..: (11.984075 N, 1.336525 W) / 0 km

Time frame............: 2020/7/7 to 2020/12/10

Number of cases.......: 2291

Expected cases........: 1000.04

Observed / expected...: 2.29

Test statistic........: 608.466723

P-value...............: < 0.00000000000000001

5.Location IDs included.: KOUDOUGOU, REO, SABOU, TENADO, NANORO, SIGLE

Coordinates / radius..: (12.295035 N, 2.324108 W) / 54.81 km

Time frame............: 2021/1/5 to 2021/6/29

Number of cases.......: 26643

Expected cases........: 22141.84

Observed / expected...: 1.20

Test statistic........: 433.131465

P-value...............: < 0.00000000000000001

6.Location IDs included.: TOMA

Coordinates / radius..: (12.686276 N, 2.885188 W) / 0 km

Time frame............: 2020/8/18 to 2020/12/16

Number of cases.......: 624

Expected cases........: 200.27

Observed / expected...: 3.12

Test statistic........: 285.467128

P-value...............: < 0.00000000000000001


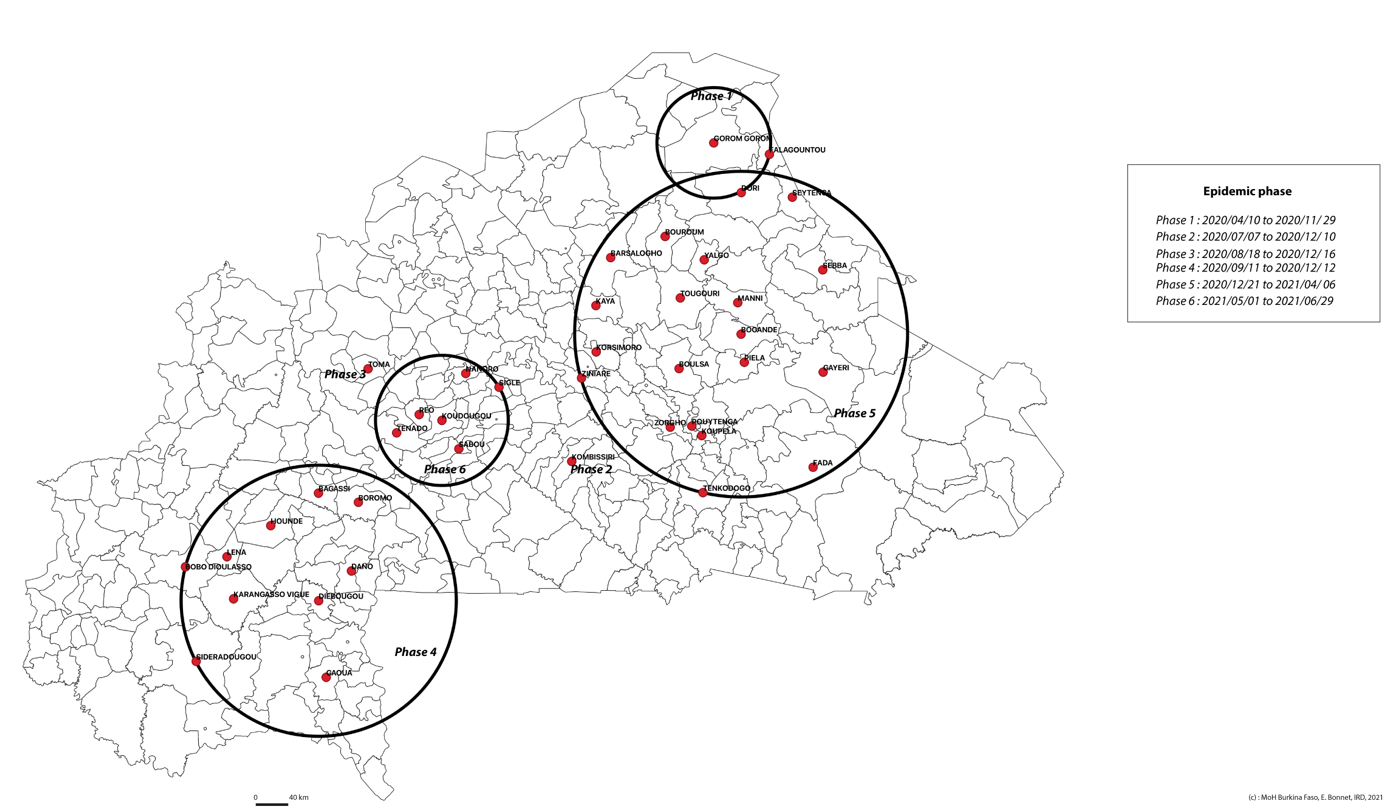


Figure 4 : Spatio-temporal cluster of COVID-19 – Burkina Faso (2020/03 – 2021/06)

**CLUSTERS DETECTED in Senegal 2020/03/01 to 2021/06/30**

1.Location IDs included.: Podor, Dagana, Richard-Toll, Pete, Keur Momar Sarr, Linguere, Dahra,

Thilogne, Sakal, Coki, Saint-Louis, Louga, Ranerou, Matam, Kebemer,

Darou Mousty

Coordinates / radius..: (16.660065 N, 14.959326 W) / 213.99 km

Time frame............: 2021/1/7 to 2021/3/14

Number of cases.......: 132626

Expected cases........: 84043.04

Observed / expected...: 1.58

Test statistic........: 12169.413198

P-value...............: < 0.00000000000000001

2.Location IDs included.: Passy, Sokone, Makacoulibantang, Foundiougne, Kaolack, Ndoffane,

Fatick, Guinguineo, Dioffor, Diakhao, Niakhar, Birkilane, Nioro du

Rip, Gossas, Joal, Thiadaye, Diourbel, Kaffrine, Bambey, Mbour

Coordinates / radius..: (13.986558 N, 16.261040 W) / 83.86 km

Time frame............: 2021/1/22 to 2021/3/14

Number of cases.......: 167426

Expected cases........: 118278.36

Observed / expected...: 1.42

Test statistic........: 9288.495707

P-value...............: < 0.00000000000000001

3.Location IDs included.: Mbake, Touba

Coordinates / radius..: (14.792911 N, 15.907353 W) / 11.50 km

Time frame............: 2020/3/11 to 2020/7/21

Number of cases.......: 37771

Expected cases........: 19518.36

Observed / expected...: 1.94

Test statistic........: 6717.690930

P-value...............: < 0.00000000000000001

4.Location IDs included.: Velingara, Medina Yoro Foula, Tambacounda, Kolda, Koumpentoum,

Khoungheul, Salemata, Dianke Makha, Sedhiou, Malem Hoddar,

Bounkiling, Goudiry

Coordinates / radius..: (13.083355 N, 14.048129 W) / 179.66 km

Time frame............: 2020/4/10 to 2020/6/18

Number of cases.......: 12791

Expected cases........: 4987.54

Observed / expected...: 2.56

Test statistic........: 4249.370690

P-value...............: < 0.00000000000000001

5.Location IDs included.: Popenguine

Coordinates / radius..: (14.628358 N, 17.075243 W) / 0 km

Time frame............: 2020/10/16 to 2021/1/2

Number of cases.......: 28375

Expected cases........: 22407.54

Observed / expected...: 1.27

Test statistic........: 735.870287

P-value...............: < 0.00000000000000001

6.Location IDs included.: Pout

Coordinates / radius..: (14.773743 N, 17.059550 W) / 0 km

Time frame............: 2020/4/27 to 2020/6/8

Number of cases.......: 2022

Expected cases........: 812.36

Observed / expected...: 2.49

Test statistic........: 634.364719

P-value...............: < 0.00000000000000001


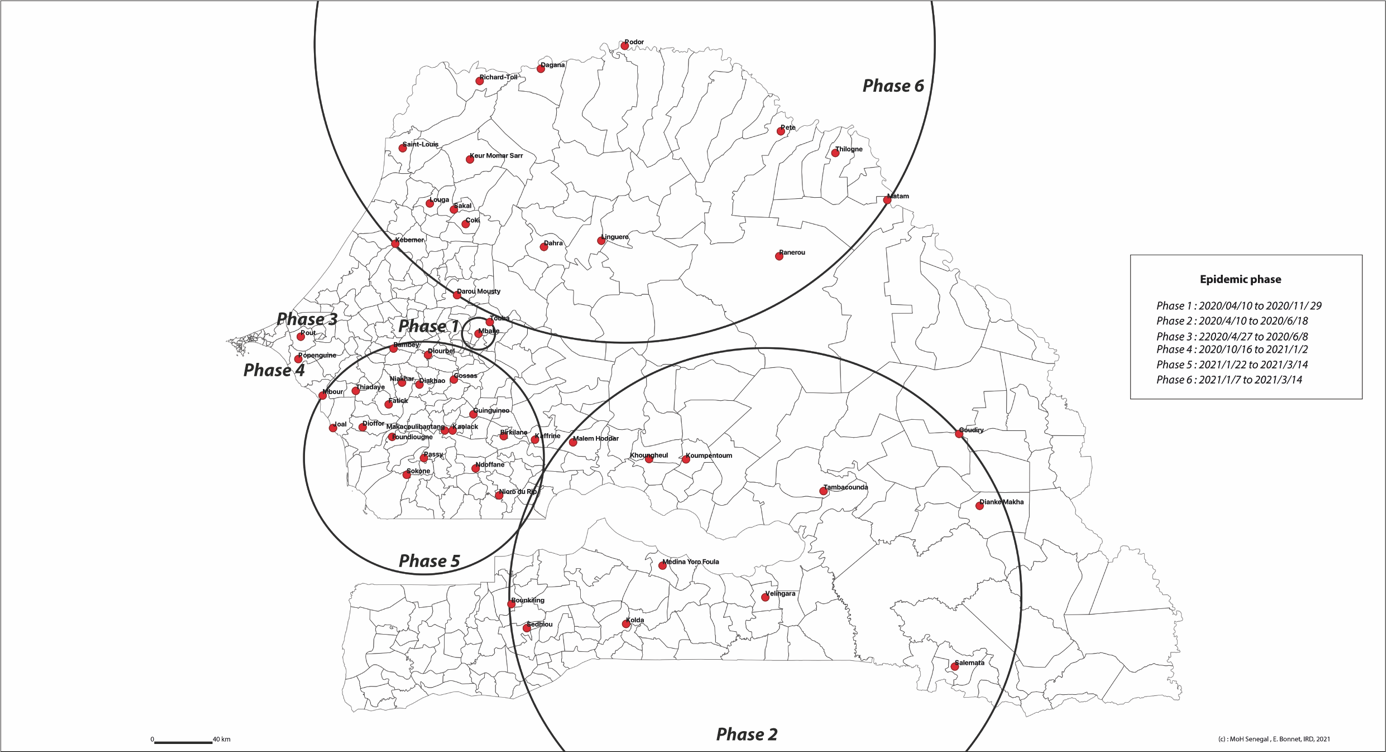


Figure 4 : Spatio-temporal cluster of COVID-19 – Senegal (2020/03 – 2021/06)
