# Supplementary material for: The COVID-19 pandemic in francophone West Africa: from the first cases to responses in seven countries
Source: BMC Public Health. 2021 Aug 2;21:1490. doi: 10.1186/s12889-021-11529-7 (PMC8327893; doi:10.1186/s12889-021-11529-7)
Supplement: Supplementary file 4 [file 12889_2021_11529_MOESM1_ESM.docx]

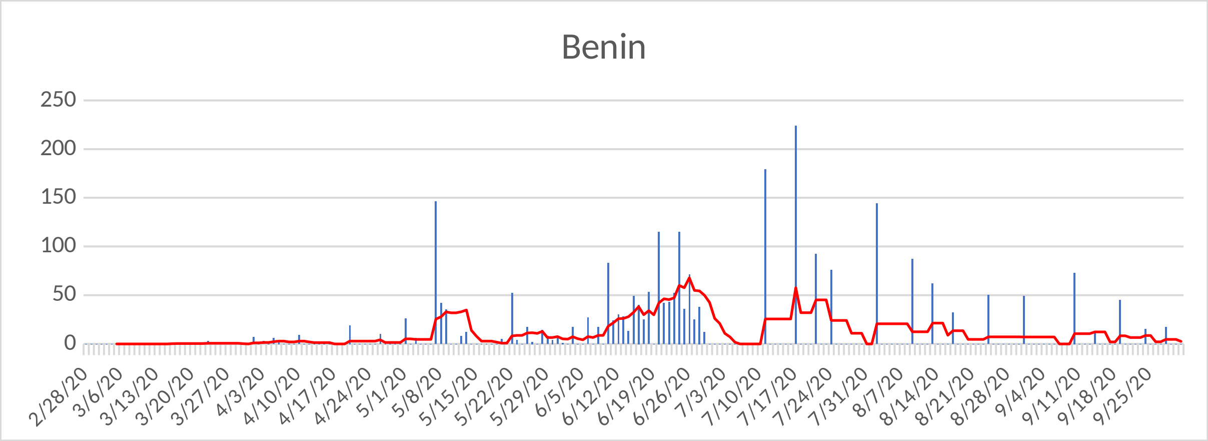
Benin
250
200
150
100
50
0
2/28/20 3/6/20 3/13/20 3/20/20 3/27/20 4/3/20 4/10/20 4/17/20 4/24/20 5/1/20 5/8/20 5/15/20 5/22/20 5/29/20 6/5/20 6/12/20 6/19/20 6/26/20 7/3/20 7/10/20 7/17/20 7/24/20 7/31/20 8/7/20 8/14/20 8/21/20 8/28/20 9/4/20 9/11/20 9/18/20 9/25/20

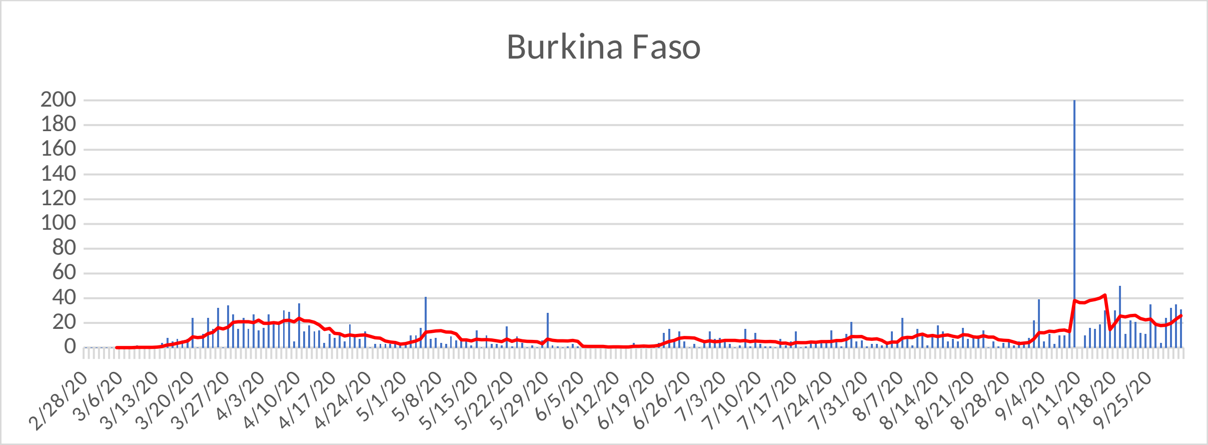
Burkina Faso
200
180
160
140
120
100
80
60
40
20
0
2/28/20 3/6/20 3/13/20 3/20/20 3/27/20 4/3/20 4/10/20 4/17/20 4/24/20 5/1/20 5/8/20 5/15/20 5/22/20 5/29/20 6/5/20 6/12/20 6/19/20 6/26/20 7/3/20 7/10/20 7/17/20 7/24/20 7/31/20 8/7/20 8/14/20 8/21/20 8/28/20 9/4/20 9/11/20 9/18/20 9/25/20

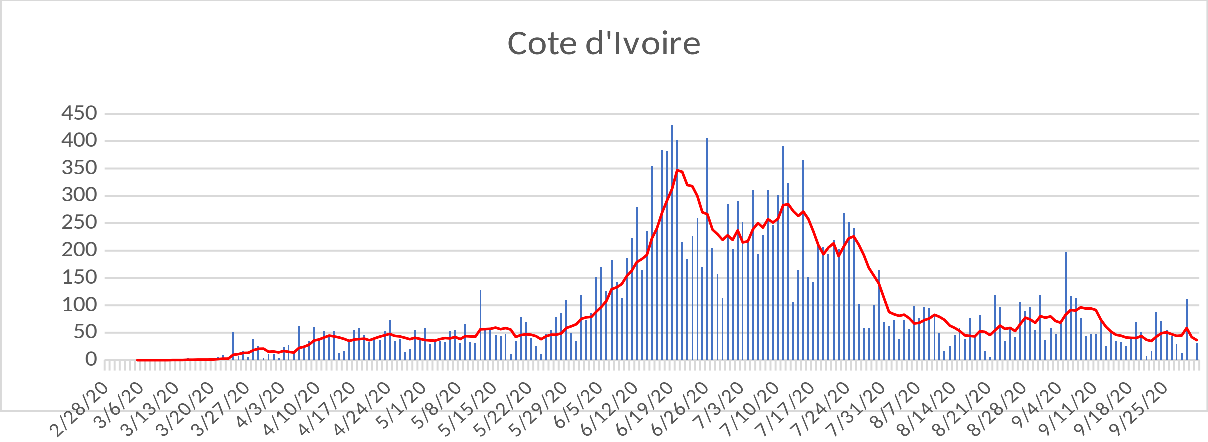
Cote d'Ivoire
450
400
350
300
250
200
150
100
50
0
2/28/20 3/6/20 3/13/20 3/20/20 3/27/20 4/3/20 4/10/20 4/17/20 4/24/20 5/1/20 5/8/20 5/15/20 5/22/20 5/29/20 6/5/20 6/12/20 6/19/20 6/26/20 7/3/20 7/10/20 7/17/20 7/24/20 7/31/20 8/7/20 8/14/20 8/21/20 8/28/20 9/4/20 9/11/20 9/18/20 9/25/20

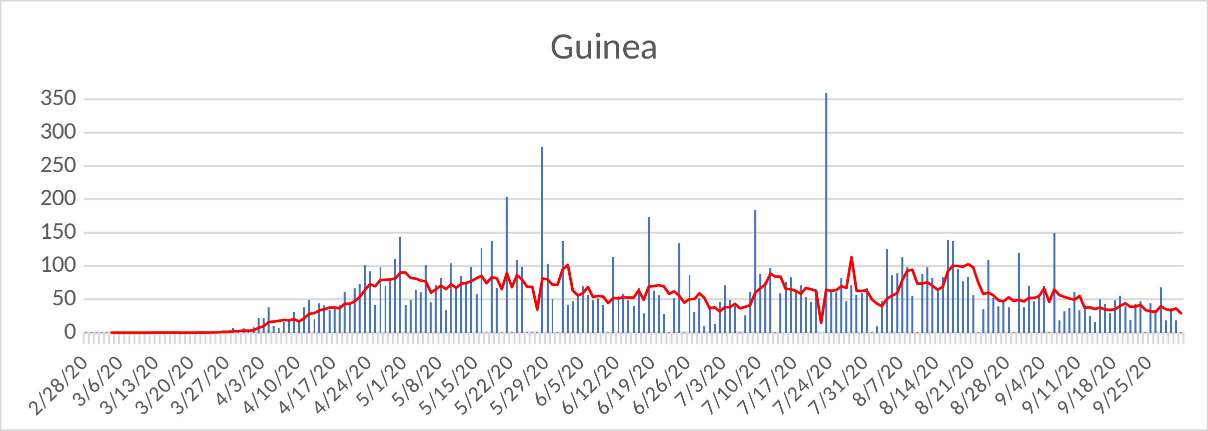
Guinea
350
300
250
200
150
100
50
0
2/28/20 3/6/20 3/13/20 3/20/20 3/27/20 4/3/20 4/10/20 4/17/20 4/24/20 5/1/20 5/8/20 5/15/20 5/22/20 5/29/20 6/5/20 6/12/20 6/19/20 6/26/20 7/3/20 7/10/20 7/17/20 7/24/20 7/31/20 8/7/20 8/14/20 8/21/20 8/28/20 9/4/20 9/11/20 9/18/20 9/25/20

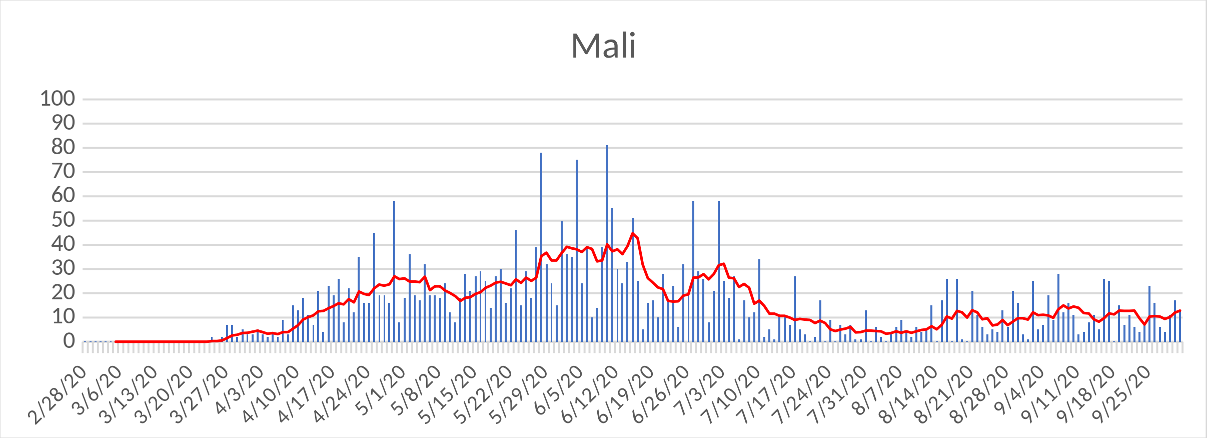
Mali
100
90
80
70
60
50
40
30
20
10
0
2/28/20 3/6/20 3/13/20 3/20/20 3/27/20 4/3/20 4/10/20 4/17/20 4/24/20 5/1/20 5/8/20 5/15/20 5/22/20 5/29/20 6/5/20 6/12/20 6/19/20 6/26/20 7/3/20 7/10/20 7/17/20 7/24/20 7/31/20 8/7/20 8/14/20 8/21/20 8/28/20 9/4/20 9/11/20 9/18/20 9/25/20

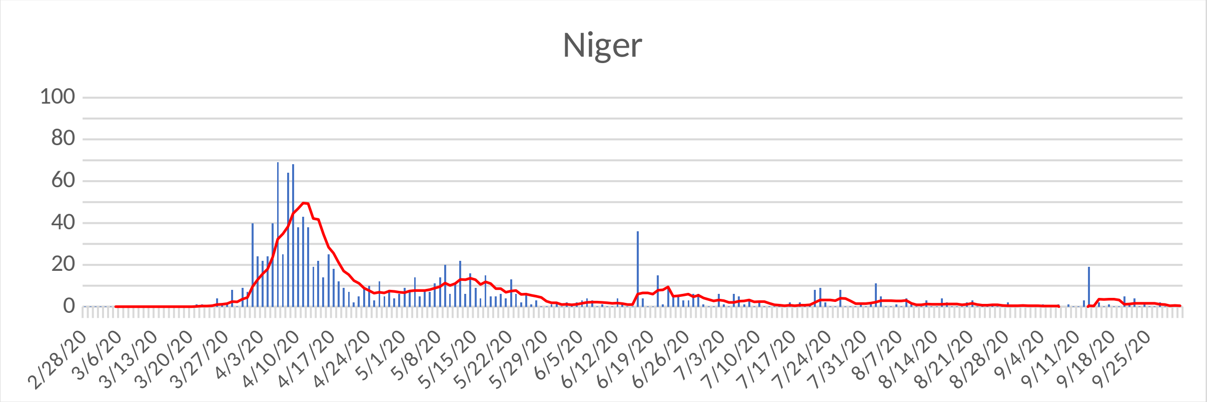
Niger
100
80
60
40
20
0
2/28/20 3/6/20 3/13/20 3/20/20 3/27/20 4/3/20 4/10/20 4/17/20 4/24/20 5/1/20 5/8/20 5/15/20 5/22/20 5/29/20 6/5/20 6/12/20 6/19/20 6/26/20 7/3/20 7/10/20 7/17/20 7/24/20 7/31/20 8/7/20 8/14/20 8/21/20 8/28/20 9/4/20 9/11/20 9/18/20 9/25/20

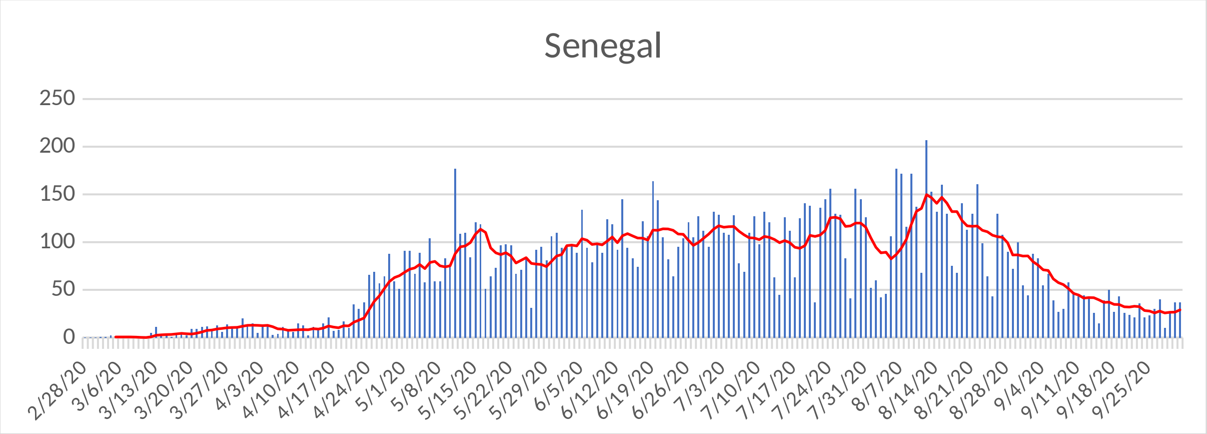
Senegal
250
200
150
100
50
0
2/28/20 3/6/20 3/13/20 3/20/20 3/27/20 4/3/20 4/10/20 4/17/20 4/24/20 5/1/20 5/8/20 5/15/20 5/22/20 5/29/20 6/5/20 6/12/20 6/19/20 6/26/20 7/3/20 7/10/20 7/17/20 7/24/20 7/31/20 8/7/20 8/14/20 8/21/20 8/28/20 9/4/20 9/11/20 9/18/20 9/25/20
